# Structural analysis of PpSP15 and PsSP9 sand fly salivary proteins designed with a self-cleavable linker as a live vaccine candidate against cutaneous leishmaniasis

**DOI:** 10.1186/s13071-022-05437-x

**Published:** 2022-10-19

**Authors:** Mahya Sadat Lajevardi, Tahereh Taheri, Elham Gholami, Negar Seyed, Sima Rafati

**Affiliations:** 1 Department of Immunotherapy and Leishmania Vaccine Research, Pasteur Institute of Iran, Tehran, Iran; 2 Department of Medical Biotechnology, School of Medicine, Zanjan University of Medical Sciences, Zanjan, Iran

**Keywords:** Protein structural homology, Sand fly, Salivary proteins, Vaccine, Cutaneous leishmaniasis

## Abstract

**Background:**

*Leishmania* parasites are deposited in the host through sand fly bites along with sand fly saliva. Therefore, salivary proteins are promising vaccine candidates for controlling leishmaniasis. Herein, two immunogenic salivary proteins, PpSP15 from *Phlebotomus papatasi* and PsSP9 from *Phlebotomus sergenti*, were selected as vaccine candidates to be delivered by live *Leishmania tarentolae* as vector. The stepwise in silico protocol advantaged in this study for multi-protein design in *L. tarentolae* is then described in detail.

**Methods:**

All possible combinations of two salivary proteins, PpSP15 and PsSP9, with or without T2A peptide were designed at the mRNA and protein levels. Then, the best combination for the vaccine candidate was selected based on mRNA and protein stability along with peptide analysis.

**Results:**

At the mRNA level, the most favored secondary structure was PpSP15-T2A-PsSP9. At the protein level, the refined three-dimensional models of all combinations were structurally valid; however, local quality estimation showed that the PpSp15-T2A-PsSP9 fusion had higher stability for each amino acid position, with low root-mean-square deviation (RMSD), compared with the original proteins. In silico evaluation confirmed the PpSP15-T2A-PsSP9 combination as a good Th1-polarizing candidate in terms of high IFN-γ production and low IL-10/TGF-β ratio in response to three consecutive immunizations. Potential protein expression was then confirmed by Western blotting.

**Conclusions:**

The approach presented herein is among the first studies to have privileged protein homology modeling along with mRNA analysis for logical live vaccine design-coding multi-proteins.

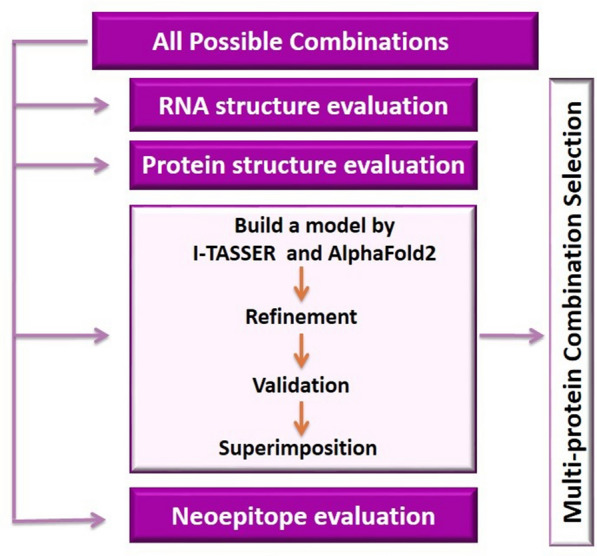

**Supplementary Information:**

The online version contains supplementary material available at 10.1186/s13071-022-05437-x.

## Background

Leishmaniasis is a vector-borne disease caused by protozoan parasites of the genus *Leishmania* and is transmitted by the female sand fly while feeding on vertebrate blood [1]. Among the main clinical manifestations, including cutaneous leishmaniasis (CL), mucocutaneous leishmaniasis (MCL), and visceral leishmaniasis (VL), CL is the most common form, with a higher prevalence rate reported in Afghanistan, Colombia, Syria, Algeria, Brazil, and Iran [2]. In Iran, CL is mainly caused by *Leishmania major* and *Leishmania tropica* parasite species. Unfortunately, despite many efforts, there is still no efficient vaccine against human leishmaniasis and disease control strategies are limited [3]. However, cured or asymptomatic individuals are the proof of concept that vaccine is hope, not hype. Various approaches, including live, live attenuated, killed, and subunit vaccines (either recombinant proteins or nucleic acid platforms) have been under intensive investigation for decades. Among all strategies examined thus far, live nonpathogenic vaccines are more promising in addition to live leishmanization [4, 5].

In recent years, sand fly saliva has been revealed to add more complexities to host–parasite interactions [6]. In fact, all types of *Leishmania* are transmitted to the host by the bite of female infected sand flies (*Phlebotomus* species in the Old World). During the blood feeding process, sand fly saliva is deposited together with parasites in the host skin [7]. Arthropod saliva is composed of a number of secreted proteins with immunomodulatory effects. Some of the protein components have been proven to be protective against leishmaniasis by inducing a delayed-type hypersensitivity response (DTH) with a T helper (Th)1-type profile [8, 9]. Hence, sand fly salivary proteins are now proposed as a component of candidate vaccines, alone or together with *Leishmania* antigens.

The main objective of the current study was to design a nonpathogenic *Leishmania tarentolae*-based live vaccine with two salivary proteins as candidates: PpSP15 from *Phlebotomus papatasi* and PsSP9 from *Phlebotomus sergenti* using in silico modeling (*Ph. papatasi* and *Ph. sergenti* are the main vectors of *Leishmania* parasites in many countries [10]). Live nonpathogenic *L. tarentolae* as a vector delivers antigens of interest and mimics live infection [11, 12]. To facilitate concurrent delivery of both proteins into one antigen-presenting cell (APC), the virus-derived 2A peptide (*Thosea asigna* virus 2A peptide sequence [T2A]) was used in between. The 2A peptides are small *cis*-acting hydrolase elements inserted between protein-coding sequences in a single open reading frame (ORF) and result in co-expression of discrete proteins at the same time [13]. The possible combinations of the two proteins with or without T2A were analyzed based on RNA and protein structures by bioinformatics tools and further evaluated by virtual immune stimulation. The combination with more favored parameters was then selected to be synthesized for future in vitro and in vivo studies.

## Methods

### Sequence retrieval

The sequences of two vaccine candidates used in this study, PpSP15 and PsSP9, were received in VR2001 and VR1020 plasmids as a gift from Dr. Jesus G. Valenzuela (Vector Molecular Biology Section, Laboratory of Malaria and Vector Research, National Institute of Allergy and Infectious Diseases, National Institutes of Health, Rockville, MD, USA). Genes were codon-optimized (https://eu.idtdna.com/codonopt) for optimal expression in *L. tarentolae*. The T2A linker sequence used between the proteins was derived from the *Thosea asigna* virus (Accession no. JA365321.1). For protein secretion purposes, the signal sequence of *Leishmania mexicana* secreted acid phosphatase (LMSAP1) was applied from the LEXSYcon2 Expression Kit’s manual (Jena Bioscience). Different combinations of the two proteins with T2A linker (PpSP15-T2A-PsSP9 and PsSP9-T2A-PpSP15) or without (PpSP15–PsSP9 and PsSP9–PpSP15) were then analyzed for further structural stability at the messenger RNA (mRNA) and protein levels. Nucleic acid and protein sequences of all the components used in this vaccine design are given in Additional file 1: Table S1.

### mRNA secondary structure prediction

Before using any software for secondary structure prediction, full-sized mRNA is needed. Since the vaccine candidate designed here will be finally utilized in a live *L. tarentolae* formulation, we needed to reach the full-sized mRNA synthesized in *L. tarentolae*. To this end, we determined the trans-splicing sites of the two 5′ untranslated region (Utr1) and 3′ untranslated region (Utr2) flanking the insertion site in the pLEXY-neo2 vector. For this, the first AG dinucleotide after the longest poly-pyrimidine tract in Utr1 (untranslated region of *Leishmania* adenine phosphoribosyl transferase [*aprt*]) was identified as a *trans*-splicing acceptor site [14]. The AG dinucleotide and the upstream sequences were cleaved to add a short consensus splice leader (SL) sequence (5′-aactaacgct atataagtatcagtttctgtactttattg-3′) as a cap for each transcript [15]. Utr2 (an intergenic region from the calmodulin operon of *Leishmania*) has a splice acceptor site for the downstream marker gene and also the polyadenylation site. The splice acceptor site in Utr2 was determined as in Utr1, and then the TA dinucleotide 500 nucleotides upstream of the acceptor was considered as the polyadenylation site [16]. Finally, a poly-A tail of 12 adenines was added to each transcript (Utr1/2 sequences and their pertinent splice and polyadenylation sites are given in Additional file 1: Fig. S1). The secondary structures of the full-sized mRNAs derived from all combinations were then predicted by the RNAfold web server (http://rna.tbi.univie.ac.at/cgi-bin/RNAWebSuite/RNAfold.cgi). RNAfold predicts both the optimal secondary and centroid structures based on their minimum free energy (MFE), and the ensemble diversity and free energy of the thermodynamic ensemble. The optimal secondary structure is predicted by the number, composition, and order of nucleotides in the RNA sequence. As a rule, a more reliable secondary structure has a lower MFE [17]. The centroid structure is the secondary structure with minimal base pair distance to all other secondary structures in the Boltzmann ensemble [18] and ensemble diversity in the Boltzmann ensemble is the average base-pair distance between all structures [19].

### Protein tertiary structure analysis by a homology modeling approach

#### Modeling and refinement of the three-dimensional (3D) protein structures

The amino acid sequence of all possible combinations was submitted to the I-TASSER server (http://zhanglab.ccmb.med.umich.edu/I-TASSER). The software generates 3D models from the amino acid sequences based on iterative threading assembly simulations and clusters all possibilities in five top models. The validity of each cluster is measured by a confidence score or *C*-score (ranging between −5 and 2). A model with a higher *C*-score almost represents a more confident model (estimated based on the significance of threading template alignment) [20]. The best 3D model of I-TASSER with a higher *C*-score was then refined by the GalaxyRefine server (http://galaxy.seoklab.org/) to reach an energy-minimized model. The server is based on the CASP10 refinement method to reconstruct all side chains of amino acids and repack them by molecular dynamics simulation at the whole protein structure level [21].

#### Validation of 3D protein structures

The refined 3D model of each construct was submitted to the ProSA-web, PROCHECK, ERRAT, verify3D, and QMEAN servers for structural validation. The ProSA-web server (https://prosa.services.came.sbg.ac.at/prosa.php) calculates the *Z*-score for an input structure and compares it with the *Z*-score of naïve proteins of the same size and known 3D structures. The *Z*-score of a protein is defined as the energy separation between the native fold and the average of an ensemble of misfolds [22]. *Z*-scores outside the peculiar range for native proteins show likely errors in the predicted structure [23]. The PROCHECK server (https://services.mbi.ucla.edu/PROCHECK/) checks the stereochemical quality of a protein structure by analyzing residue by residue geometry and plots the phi-psi torsion angles for each residue of the protein in allowed and disallowed regions (Ramachandran plots) [24]. The ERRAT server (http://services.mbi.ucla.edu/ERRAT/) analyzes non-bond atom–atom interactions compared with well-refined structures [25]. The verify3D (http://services.mbi.ucla.edu/Verify_3D) detects the compatibility of a 3D model with its own amino acid sequence in comparison with proper structures and by assigning a structural class based on its environment (alpha, beta, loop, polar, and nonpolar) and location [26]. QMEAN describes major local geometrical aspects of protein structures. The local geometry is analyzed by torsion angle potential over three consecutive amino acids (https://swissmodel.expasy.org/qmean/) [27].

#### Structural superimposition

All the validated three-dimensional models were compared with original proteins (already modeled and validated by the same approach) using UCSF (University of California, San Francisco) Chimera v1.15 software (https://www.cgl.ucsf.edu/chimera/). The program makes a fit after detecting residues that should be paired. For this, the software utilizes secondary structures and sequences to overlap similar structures even with low similarity [28]. The result is evaluated using the root-mean-square deviation (RMSD) of atomic positions. Typically, RMSD is used as a quantitative measure of similarity between two or more protein structures. A lower RMSD indicates a higher similarity between the two structures. For closely homologous proteins, the RMSD is as small as 3 Å [29].

### Helper T lymphocyte (HTL) epitope prediction

Major histocompatibility complex class II (MHC-II) binders or helper T lymphocyte (HTL) epitopes were predicted using a combination of motif-based (SYPHPEITY), machine learning (IEDB and NetMHCIIpan), and PSSM (RANKPEP) algorithms in the context of human alleles. SYFPEITY (http://www.syfpeithi.de/) is a matrix-based method derived from over 7000 known MHC binders [30]. Peptides scored above 20 were chosen as promising epitopes. The IEDB server (http://tools.iedb.org/mhcii/) provides different machine learning algorithms. The “IEDB recommended method” was selected for peptide prediction using a reference set of 27 human alleles (full human leukocyte antigen [HLA] reference set with a population coverage over 97%) [31]. Epitope selection was based on the calculated percentile ranks. For each peptide, a percentile rank is generated by comparing the peptide’s score against the scores of 5 million random 15-mers selected from the SWISS-PROT database. A small numbered percentile rank indicates higher affinity. Percentile ranks below 5% were used to select binders. RANKPEP (http://imed.med.ucm.es/Tools/rankpep.html) is a server based on position-specific scoring matrices (PSSMs) to predict MHC-II binding. PSSMs in RANKPEP are associated with a specific binding threshold (PSBT) above which epitopes are sorted [32]. For each selected allele, the PSBT is determined based on a consensus epitope with an optimal score. The NetMHCIIpan-4.0 (http://www.cbs.dtu.dk/services/NetMHCIIpan/) server predicts MHC-II-binding epitopes using artificial neural networks (ANNs). Epitopes scored under threshold values (5% percentile rank) were selected as binders [33]. Epitopes confirmed using at least two different servers were selected for further interferon-gamma (IFN-γ) induction analysis by IFNepitope (http://crdd.osdd.net/raghava/ifnepitope/). The server uses a dataset of IFN-γ-inducing and non-inducing MHC-II binders and predicts binders with different machine learning, motif-based and hybrid approaches. The highest level of accuracy is achieved using the hybrid model (> 81.39%). The selection threshold was set below 0.5.

### Cytotoxic T lymphocyte (CTL) epitope prediction

Several servers, including NetCTL 1.2, IEDB, RANKPEP, and SYPHPEITY, were used to predict CTL epitopes (9- and 10-mers in length) for 12 human MHC-I supertypes. NetCTL 1.2 (http://www.cbs.dtu.dk/services/NetCTL/) predicts CTL epitopes based on MHC-I affinity, proteasomal cleavage, and transport-associated protein (TAP) binding efficiency using the ANN method and the weight matrix [34]. The cutoff score for epitope prediction was set at 0.75. IEDB (http://tools.iedb.org/processing/) also predicts CTL epitopes by combining proteasomal cleavage, TAP, and MHC-I binding by the “recommended” method. Peptides with half maximal inhibitory concentration (IC_50_) values less than 50 nM are ranked as strong binders, and IC_50_ scores between 50–500 nM indicate intermediate MHC binding affinity [35]. Rankpep (http://imed.med.ucm.es/Tools/rankpep.html) predicts proteosomal cleavage in addition to MHC-I-binding affinity using position-specific scoring matrices (PSSMs) [31]. The top-scored epitopes are selected based on PSBT. SYFPEITY (http://www.syfpeithi.de/) scores MHC-I binders based on the binders’ matrix, with top binders scoring over 20 [33]. Peptides scored as binders using at least two servers were selected as promising epitopes.

### Evaluation of population coverage

Population coverage of the predicted epitopes was evaluated with the IEDB population coverage tool (http://tools.iedb.org/population/) based on the MHC allele distribution [36]. We focused on the areas where the most new CL cases occurred according to the World Health Organization (WHO) in 2019 (https://www.who.int/news-room/fact-sheets/detail/leishmaniasis). Calculations were performed for the combination of HTL and CTL epitopes.

### Tertiary structure validation of candidate construct using AlphaFold2 server

The amino acid sequence of the selected construct as vaccine candidate was submitted to the https://colab.research.google.com/github/sokrypton/ColabFold/blob/main/AlphaFold2.ipynb server (AlphaFold2) for further validation [37, 38]. The server predicts five top-ranked 3D structures with almost experimental accuracy based on multiple-sequence analysis (the first rank is the most preferred one). It combines numerous novel neural networks and training procedures relying on the evolutionary, geometric, and physical constraints of protein structures to improve prediction accuracy. The prediction quality is estimated based on two confidence metrics called the predicted Local Distance Difference Test (pLDDT) and Predicted Aligned Error (PAE). The pLDDT reflects local confidence in the structure and residues with pLDDT ≥ 70 are classified as confident. The PAE confidence measure (the expected positional error) visualizes multiple sequence alignment diversity and is interpreted by color codes from 0–30. The lower values (close to zero and colored in blue), indicate higher prediction quality. The higher values (close to 30 and colored in red), indicate lower prediction quality.

### Physicochemical parameters, antigenicity, and allergenicity of the constructs

The ProtParam web server (http://web.expasy.org/protparam/) was used to define the most important physicochemical properties of the candidate vaccine construct, including theoretical pI (isoelectric point), molecular weight, instability index, and grand average of hydropathicity (GRAVY) [39]. VaxiJen v2.0 and ANTIGENpro servers were advantaged for antigenicity prediction of the selected vaccine construct. The prediction by VaxiJen v2.0 (http://www.ddg-pharmfac.net/vaxijen/VaxiJen/VaxiJen.html) is based on the physicochemical properties of proteins. Depending on the target organism, the server accuracy varies between 70 and 89% [40]. ANTIGENpro (http://scratch.proteomics.ics.uci.edu/) is a sequence-based and alignment-free predictor. The server utilizes microarray data files to predict protein antigenicity independent of the pathogen type [41]. The AllerTOP v2.0 server (http://www.ddgpharmfac.net/AllerTOP/) was used to evaluate the allergenicity of the vaccine construct. The server uses an algorithm based on auto and cross-covariance transformation and KNN (the *k*-nearest neighbors) methods to categorize protein sequences. The algorithm is trained by a set of 2427 allergens and 2427 nonallergens. The prediction accuracy of the server has been reported to be 85.3% [42].

### Virtual immune simulation

To characterize the immunogenicity of the candidate vaccine construct, in silico immune simulations were performed using the C-ImmSim (http://150.146.2.1/C-IMMSIM/index.php) server [43]. This agent-based simulator employs PSSM and machine learning techniques to predict cellular and humoral immune responses induced by an antigen in humans. The C-ImmSim server predicts the immune responses consistent with prior experimental studies. The PpSP15-T2A-PsSP9 vaccine candidate and PpSP15-T2A and PsSP9-His-tag were injected three times, at time steps 1, 42, and 84, at 2-week intervals (recommended by Kaba et al. [44].). Each time step is equal to 8 h, reflecting a cell division cycle in the real environment. Thus, the first injection occurs in time step 1 at day 0, the second injection in time step 42 at day 14, and the third injection in time step 84 at day 28. All simulation parameters were saved as default except for the HLA alleles, which were set on heterozygote expression of the most frequent alleles in the Iranian population. Cytokine production and T helper cell polarization and diversity were analyzed. The Simpson or D index was used as a measure of diversity. The lower values of the Simpson index (closer to zero) correlate with greater diversity and higher emergence of various dominant clones of T cells.

### Gene cloning in *L. tarentolae* and expression confirmation

To meet the final goal of vaccination, the candidate construct must be cloned in pLEXSY vectors. These plasmids are professionally advantaged for stable transfection in the *L. tarentolae* parasite. The selected construct was synthesized and delivered in pMA-RQ plasmid by Thermo Fisher Scientific Inc. (USA). The pLEXSY-neo2 vector (Jena Bioscience, Germany) was used as an integrative vector for stable genome transfection using *Bgl*II (5′ end) and *Nhe*I (3′ end) restriction enzymes (Fermentas, USA) to sub-clone the target sequence. The recombinant vector was linearized by *Swa*I digestion and then transfected into *L. tarentolae* by a pre-set protocol [45]. Stable *L. tarentolae* recombinants co-expressing PpSP15 and PsSP9 were cultured in M199 supplemented with 5% heat-inactivated fetal bovine serum (FBS). Five days later, the culture supernatant was harvested and mixed with Ni-NTA agarose beads (QIAGEN, Germany) and incubated overnight to capture the secreted salivary proteins. Beads with adsorbed protein were then mixed with sample buffer and loaded on 17.5% sodium dodecyl sulfate–polyacrylamide gel electrophoresis (SDS-PAGE) gel to further confirm the protein expression by anti-His-tag (QIAGEN, Germany) Western blotting.

## Results

### Sequence retrieval and construct design

**Fig. 1:**
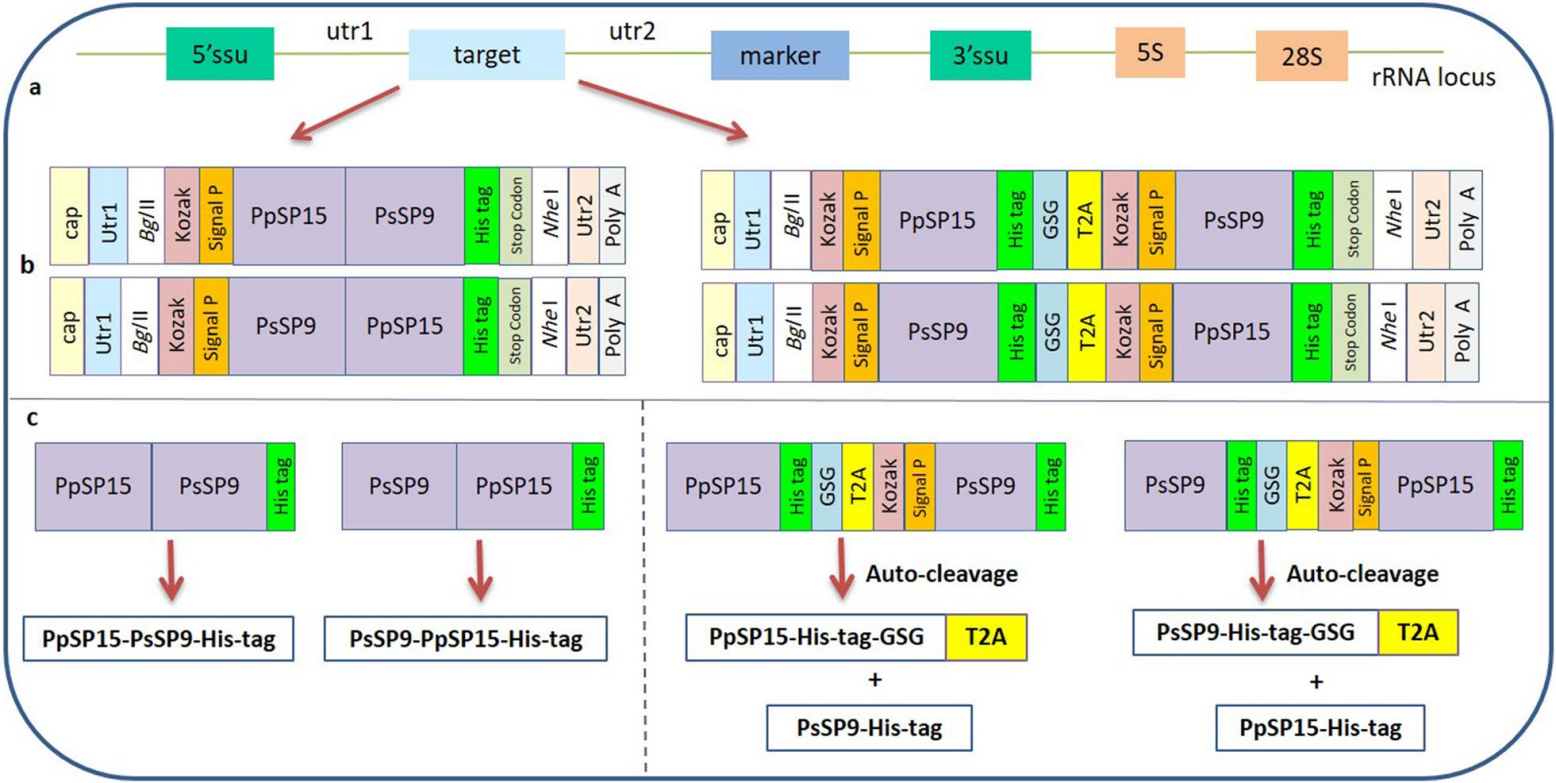
RNA and protein sequences of all studied constructs. The target gene is inserted by stable transfection into the rRNA locus of *L. tarentolae*. **a** Schematic presentation of recombinant *L. tarentolae* rRNA locus. **b** Schematic presentation of all possible PpSP15/PsSP9 combinations with and without T2A at the mRNA level. Cap (SL sequence), Utr1 spliced sequence, Kozak, and signal sequences were N-terminally inserted in addition to the C-terminal His-tag, stop codon, Utr2 spliced sequence, and poly-A tail. **c** Schematic presentation of all possible PpSP15/PsSP9 combinations with and without T2A at the protein level. PpSP15 and PsSP9 fusions with the T2A linker give rise to separate individual proteins tagged with C-terminal T2A or six histidines accordingly

In this study, four combinations of the two sand fly salivary sequences PsSP9 and PpSP15 with or without 2A linker peptide were analyzed at the mRNA and protein levels to choose the most structurally reliable combination as a live candidate vaccine. Figure 1a depicts the expression cassette integrated into *L. tarentolae* 18S rRNA locus. Figure 1b illustrates all possible mRNA sequences transcribed from transgenic *L. tarentolae* either fused directly without a linker (PpSP15–PsSP9 and PsSP9–PpSP15) or with an autocleavable 2A peptide inserted between the two ORFs (PpSP15-T2A-PsSP9 and PsSP9-T2A-PpSP15). GSG linker upstream of the 2A peptide is used to increase the cleavage efficiency. In all constructs, Cap (SL sequence), Utr1 spliced sequence, Kozak, and signal sequences were N-terminally inserted in addition to the C-terminal His-tag, stop codon, Utr2 spliced sequence, and poly-A tail (Fig. 1b). *Bgl*II and *Nhe*I restriction sites were inserted, respectively, at the 5′ and 3′ ends of each construct for further cloning in the pLEXSY-neo2 vector. Figure 1c demonstrates the fusion structures at the protein level. Constructs with self-cleavable T2A linkers generate individual T2A- or histidine-tagged (His-tag) proteins as well as the whole fusion (although less likely).

### mRNA secondary structure prediction

**Fig. 2:**
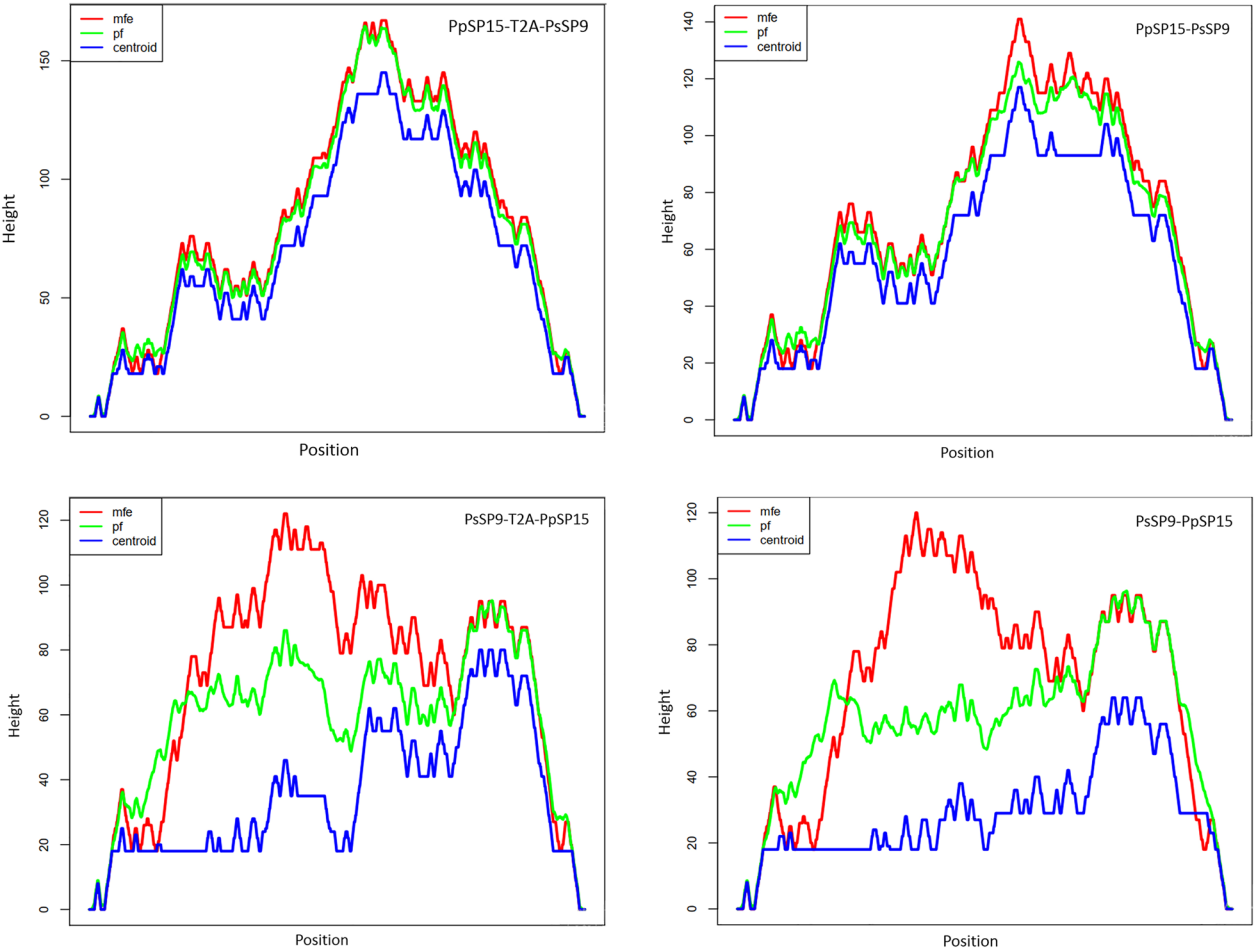
Mountain plot analysis by RNAfold for all possible combinations. The full-sized mRNA sequence of all combinations was submitted to the RNAfold web server. This server predicts two secondary structures including optimal and centroid structures. Mountain plot analysis (sequence position versus the number of base pairs that enclose that position) shows the similarity between the optimal secondary structure (red line), the centroid structure (green line), and pair probabilities (blue line)

The secondary structure of mRNA strongly affects the final outcome of in vivo expression. Bioinformatics tools help to better choose the most stable constructs based on MFE. The full-sized mRNA sequence transcribed in *L. tarentolae* was first estimated using trans-splicing and polyadenylation, and then the sequences were analyzed individually using RNAfold. Table 1 summarizes the RNAfold output for all different constructs analyzed. As indicated, PpSP15-T2A-PsSP9 generates a centroid structure with the lowest MFE in addition to lower ensemble diversity, where both parameters demonstrate more reliable prediction. RNAfold also compares the optimal secondary structure (Additional file 1: Fig. S2a) and centroid structure (Additional file 1: Fig. S2b), where higher similarity further indicates reliable prediction. As shown, PpSP15-T2A-PsSP9 presents the highest similarity between the aforementioned structures, where the MFE of both structures are the closest (Table 1) confirming that T2A insertion guarantees mRNA stability compared with the linker-free construct. Furthermore, mountain plots (a graph that plots sequence position versus the number of base pairs that enclose that position) indicate the highest similarity between the optimal secondary structure, the centroid structure, and pair probabilities for PpSP15-T2A-PsSP9 among the other combinations (Fig. 2). Therefore, at the mRNA level, PpSP15-T2A-PsSP9 was the preferred construct among all the different possibilities.

**Table 1:** RNAfold prediction results for all different combinations

Constructs	MFE of the optimal secondary structure (kcal/mol)	Ensemble diversity^a^	MFE of the centroid secondary structure^b^ (kcal/mol)	The free energy of thermodynamic ensemble^c^ (kcal/mol)
PpSP15-T2A-PsSP9	−399.80	262.68	−329.73	−423.73
PpSP15–PsSP9	−317.50	274.74	−255.10	−339.65
PsSP9-T2A-PpSP15	−402.60	402.39	−265.50	−424.09
PsSP9–PpSP15	−323.50	346.15	−186.30	−342.44

MFE: minimum free energy

^a^Is the average base-pair distance between all structures in the Boltzmann ensemble

^b^MFE of the structure with minimal base-pair distance to all structures in the thermodynamic ensemble

^c^Is a statistical ensemble in statistical equilibrium that prepares a way to infer the properties of a real thermodynamic system

### Homology modeling and validation of the 3D structures

The tertiary structure of the fusion proteins derived from each combination (Fig. 1c) was modeled using the I-TASSER server, which predicts the top five 3D models based on the *C*-score. The C-score ranges between −5 and 2, where higher values show a model with higher prediction confidence. The first model with the highest *C*-score of each construct was further refined using GalaxyRefine to minimize the energy. GalaxyRefine returns five refined models in the results. The best model was selected based on higher values of global distance test–high accuracy (GDT-HA) and Rama favored and lower values of RMSD for each possible construct (Additional file 1: Table S2). All predicted 3D models were further validated to ensure the reliability of the model from the energy points.

**Fig. 3:**
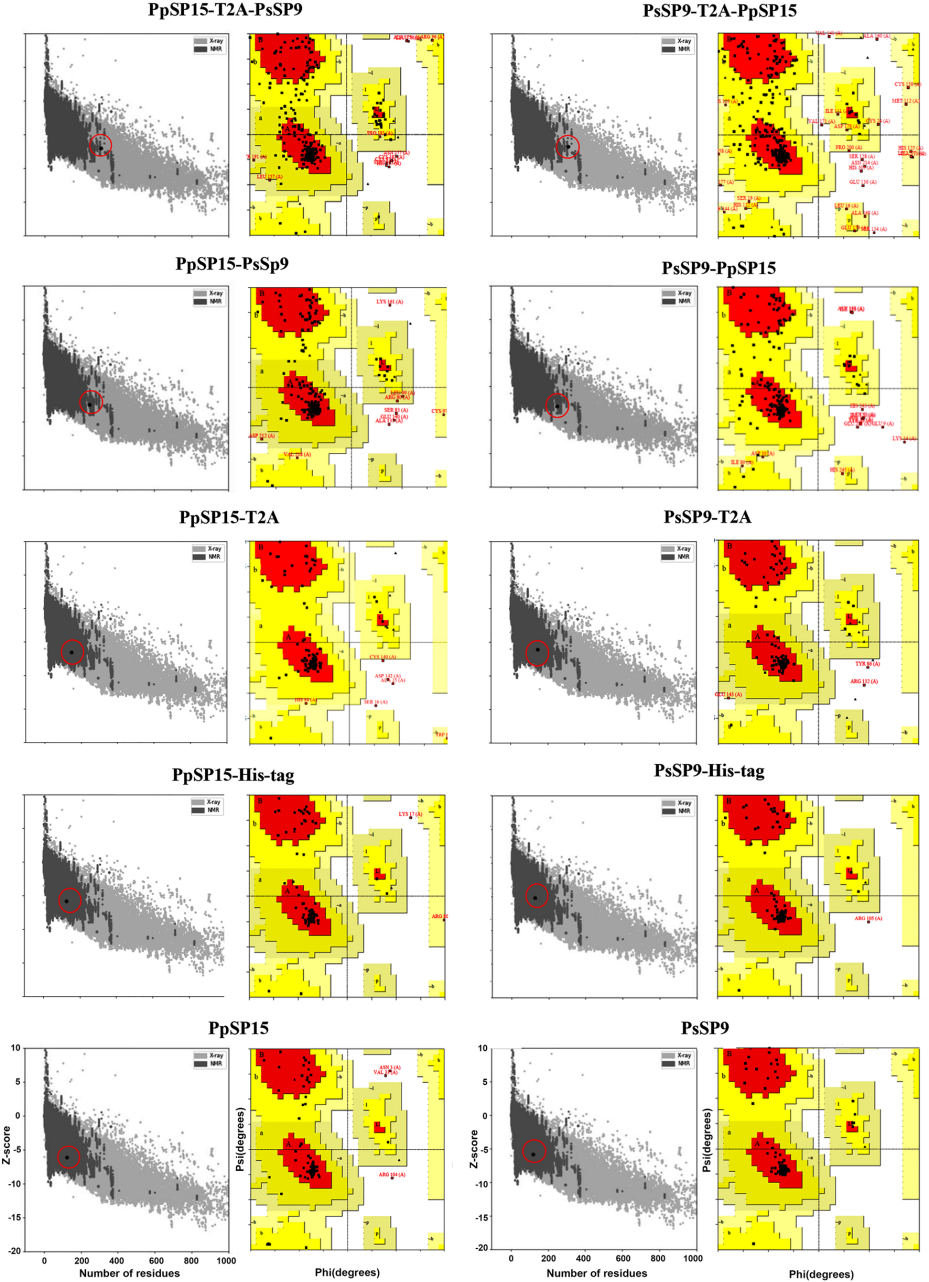
Modeling validation of all combinations using the ProSA and PROCHECK web servers. The refined model of each combination was submitted to the ProSA and PROCHECK servers for structure validation. The ProSA-web server provides *Z*-score for an entry structure and compares it with the *Z*-score of naïve proteins with known 3D structures of the same size. Left graphs represent the *Z*-score plot by ProSA, which indicates overall model quality. Black circles describe the *Z*-score of the corresponding combination. The predicted *Z*-score of all combinations was approximately between (−5) and (−8) similar to native proteins. The PROCHECK server shows the protein structure stereochemical quality by plotting the phi-psi torsion angles for each residue in the allowed and disallowed regions. Right graphs represent PROCHECK analysis of allowed vs. non-allowed atomic distance. For all models, more than 95% of residues were located in the favored and allowed regions, indicating protein stability

**Fig. 4:**
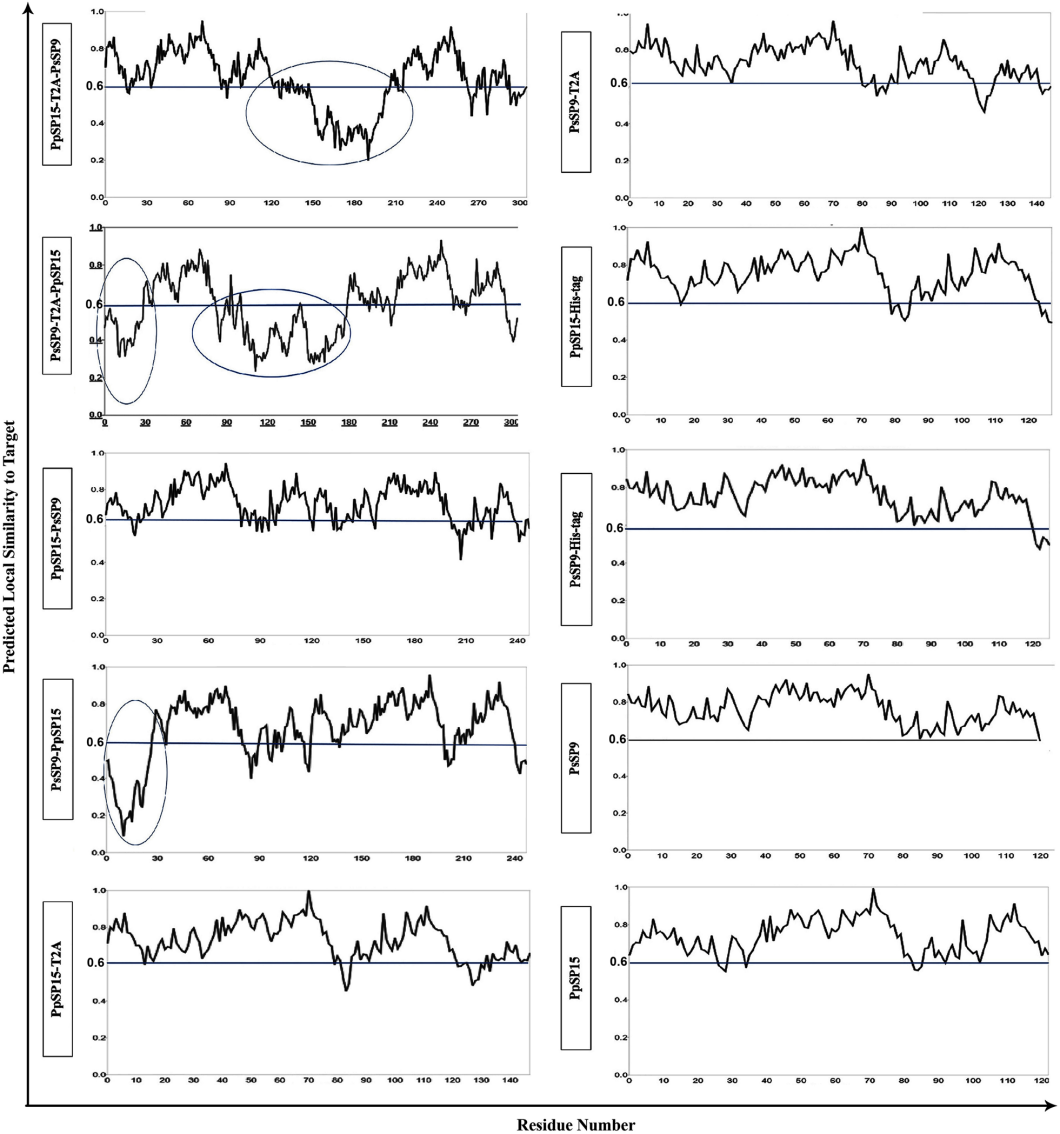
Local quality estimation analysis of all protein combinations by QMEAN analysis. The quality of all refined models was assessed using the QMEAN server. QMEAN displays the local quality score for protein structures residue by residue. Solid lines on each local quality estimation graph indicate high local stability for the corresponding amino acid position (local quality scores over 0.6). Circled areas indicate instability

A valid model is actually verified by different parameters. Many online tools are available to check the validity of models, each focused on a special aspect (Table 2). In this study, quality assessment of all the refined models by the ProSA-web server showed that all reported *Z*-scores fell within the range of naïve proteins of a similar size (Table 2 and Fig. 3). Ramachandran plots then revealed that generally more than 95% of residues of all models fell within the favored and allowed regions, which are a good indicator of protein stability (Table 2 and Fig. 3). The ERRAT server demonstrated that the overall quality factor for all models was almost above 70% (except for PsSP9-T2A-PpSP15), while the ideal overall quality factor was more than 95%. Using verify3d evaluation, more than 90% of the residues had average 3D/1D scores ≤ 0.2 for all models (good models show more than 80% 3D/1D scores over 0.2). To finalize the selection of a valid fusion model, the analysis was continued using the QMEAN SWISS-Model, which further improved the verification and selection process by the local quality score. Figure 4 displays the local quality score for all combinations in the local quality estimation graphs. As indicated, the PpSP15–PsSP9 fusion has local position scores greater than 0.6 (horizontal solid lines), which indicates high local stability for each amino acid position. In contrast, PsSP9–PpSP15 loses local stability around the N-terminus. The T2A insertion agitates local stability around T2A and the downstream signal peptide for both fused structures. However, dissociated proteins with T2A- or His-tags have local stability over 0.6 without any priority between structures. As a result, at the protein level, 5′-end PpSP15 and 3′-end PsSP9 spaced with or without a linker generate more structurally stable constructs. Furthermore, the PpSP15-T2A and the PsSP9-His-tagged dissociated components were structurally stable.

**Table 2:** Protein validation results for all combinations

Construct	Software					
	ProSA-web *Z*-score^a^	ERRAT overall quality factor^b^	Verify3D (%)^c^	Ramachandran analysis^d^		
				Favored	Allowed	Disallowed
PpSP15-T2A-PsSP9	−7.03	80.201%	92.16	74.6%	22.9%	2.5%
PpSP15–PsSP9	−7.49	89.167%	91.94	84.3%	14%	1.7%
PsSP9-T2A-PpSP15	−6.76	60.403	94.44	69.8%	27.4%	2.8%
PsSP9–PpSP15	−7.99	78.75%	95.97	81%	14.8%	4.2%
PpSP15-T2A	−6.56	74.2857	100	88	9	3
PpSP15-Histag	−5.79	94.167	100	92.5%	6.6%	0.8%
PsSP9-T2A	−6.1	93.4783	100	89.6%	8.8%	1.5%
PsSP9-Histag	−5.2	99.145	98.41	95%	4.1%	0.8%
PpSP15	−6.09	99.1304	98.37	87.8%	9.6%	2.6%
PsSP9	−5.83	97.3214	90.83	92.5%	5.8%	0

^a^Indicates the overall model quality

^b^The percentage of the proteins for which the calculated error value falls below 95% rejection limit

^c^Percentage of amino acids with a 3D/1D score ≥ 0.2

^d^Dihedral angles psi (ψ) versus phi (ϕ) distribution

### Superimposition with original proteins

The final step of homology modeling was to check the similarity of predicted models with the individual proteins using RMSD analysis (For closely homologous proteins, the RMSD is as small as 3 Å). For this, the original proteins of PpSP15 and PsSP9 (modeled and validated in parallel with other proteins) were overlaid to all different protein combinations along with *Phlebotomus duboscqi* SP15 (unlike others, only *Ph. duboscqi* SP15 (PdSP15) has an available 3D model in the PDB database and crystallographic data [4OZD]). RMSD results (Table 3) indicated that both PpSP15–PsSP9 and PsSP9–PpSP15 combinations without linker are well superimposed on all PpSP15, PsSP9, and PdSP15 proteins with low RMSDs. However, when the linker comes in between, PpSP15-T2A-PsSP9 shows some deviation toward higher RMSDs while superimposed on PsSP9 (which could be ignored) compared with PsSP9-T2A-PpSP15. As indicated in Table 3, neither the T2A sequence nor the His-tag at the C-terminus of dissociated proteins strongly affected the superimposition result (very low RMSDs). PdSP15 is structurally very similar to PpSP15, although the sequence identity is approximately 63%. Therefore, we used superimposition with this structure as the control of the modeling process. As demonstrated, PdSP15 was very well superimposed on all PpSP15 structures either within the fusion constructs or on the individual components PpSP15-T2A or PpSP15-His-tag. The Additional file 1: Fig. S3 displays all possible models superimposed on their original proteins.

**Table 3:** RMSD results of superimposition against original proteins

Construct	Superimposition		
	Pdsp15	PpSP15	PsSP9
PpSP15-T2A-PsSP9	^a^2.251	2.242	9.967
PpSP15–PsSP9	3.135	3.371	2.761
PsSP9-T2A-PpSP15	2.208	2.195	6.754
PsSP9–PpSP15	1.833	2.008	5.899
PpSP15-T2A	0.9	0.719	–
PpSP15-His-tag	0.833	0.543	–
PsSP9-T2A	–	–	0.748
PsSP9-His-tag	–	–	0.621

^a^For closely homologous proteins, the RMSD is as small as 3 Å

### T-cell epitope prediction and population coverage

One of the main drawbacks of fusing two or more proteins is that new antigens are generated at the junction region. This can eventually divert the immune response toward an irrelevant dominant epitope that compromises expected immunity. To avoid this, bioinformatics tools can help in predicting junction peptides. By means of several different servers, linker-free constructs (PpSP15–PsSP9 and PsSP9–PpSP15) were first evaluated for junctional MHC-II binding epitopes. The PsSP9–PpSP15 can raise a strong-binding HLA-II junctional epitope but not the PpSP15–PsSP9 fusion (Additional file 1: Table S3). The T2A insertion created no significant junctional epitope in either PsSP9-T2A-PpSP15 or PpSP15-T2A-PsSP9 fusions. Therefore, the PpSP15–PsSP9 fusion was superior both with and without the T2A linker. Based on all RNA and protein stability results, we further focused on PpSP15-T2A-PsSP9 dissociated components for HTL and CTL potential epitope prediction (Additional file 1: Table S4). Among all the predicted HTL epitopes, eight epitopes were selected for PpSP15-T2A and two for PsSP9-His-tag. Positive scores of these epitopes at the IFNepitope server output showed that they are potential IFN-γ inducers (Additional file 1: Table S4). Meanwhile, five CTL epitopes were selected for PpSP15-T2A and five for PsSP9-His-tag. All selected epitopes (10 CTL and 10 HTL epitopes) were then used in combination to estimate the vaccine population coverage. This candidate vaccine was found to cover 85.46% of the Iranian population. Additionally, the highest and lowest coverage were related to Saudi Arabia (93.26%) and Pakistan (42.39%), respectively (Additional file 1: Fig. S4). Thus, according to the results, this candidate vaccine could be useful in the regions with the highest rate of CL cases in the Old World.

### AlphaFold2 validation of selected candidate

**Fig. 5:**
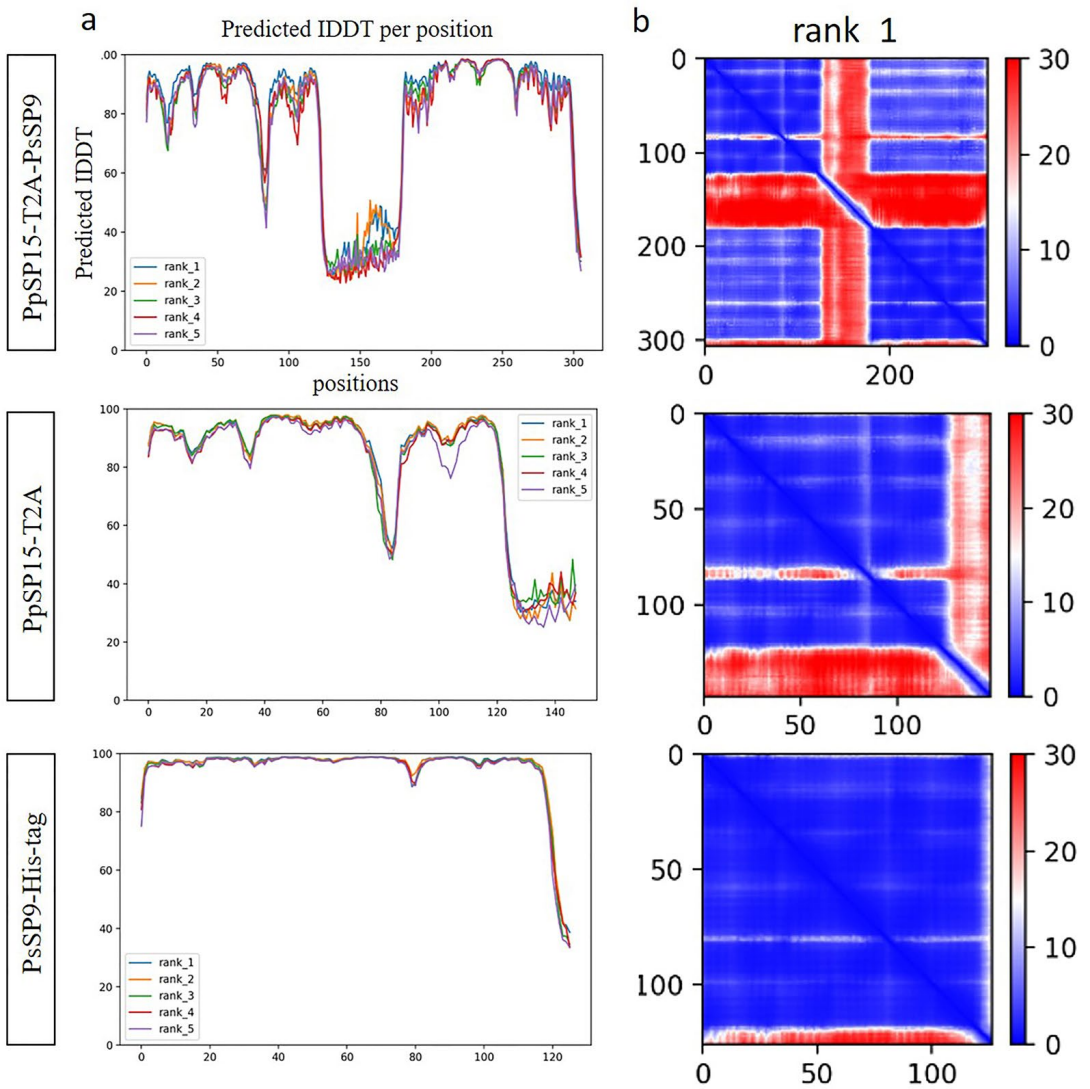
The outputs of the AlphaFold2 server for the selected vaccine candidate construct. The AlphaFold2 server provides 3D models from the amino acid sequences for the selected construct (PpSP15-T2A-PsSP9) as a whole and constituting proteins (PpSP15-T2A and PsSP9-His-tag) based on two confidence metrics: pLDDT, predicted Local Distance Difference Test **a** and PAE, Predicted Aligned Error **b**. Residues with pLDDT ≥ 70 are confident (lower values are detectable around the T2A linker). The lower PAE (around zero in blue) shows higher prediction quality

AlphaFold2 is a newly developed server that predicts the 3D models of proteins based on sequence alignments and validates the models by pLDDT and PAE criteria. To further confirm the model selected through the homology modeling approach, PpSP15-T2A-PsSP9 and its cleavage components (PpSP15-T2A and PsSP9-His-tag) were further validated using this server. It predicts five top-ranked 3D models from the amino acid sequences based on pLDDT and PAE. pLDDT values ≥ 70 and lower PAE indicate a 3D model with higher prediction confidence. As indicated in Fig. 5, almost all residues of the whole PpSP15-T2A-PsSP9 and its constituting proteins showed pLDDT over 70 (except for T2A regions), which was consistent with QMEAN local quality scores. The pLDDT value is reflected on PAE criteria, which illustrates positional errors (in red) quite comparable with agitated regions in pLDDT plots. However, QMEAN predicted PpSP15-T2A and PsSP9-His-Tag with stable values greater than 0.6. The difference between the homology modeling approach through I-TASSER prediction and the AlphaFold2 3D prediction could be the refinement step which is routinely followed after I-TASSER prediction but not by the AlphaFold2 server.

### Physicochemical properties, antigenicity, and allergenicity of the selected candidate

After the selection of PpSP15-T2A-PsSP9 as a fusion construct, the protein as a whole and as constituting proteins were analyzed by ProtParam. The protein as a whole and as PsSP9-His-tag are stable (instability index < 40) but not PpSP15-T2A (instability index > 40 similar to PpSP15). The GRAVY index (ranging from −2 to +2 for most proteins with the positively rated proteins being more hydrophobic) indicated that the proteins were hydrophilic (Additional file 1: Table S5). Moreover, the PpSP15-T2A-PsSP9 and the two resulting proteins (PpSP15-T2A and PsSP9-His-tag) were not allergenic but antigenic instead (Additional file 1: Table S6).

### In silico evaluation of the immune response against the selected candidate

**Fig. 6:**
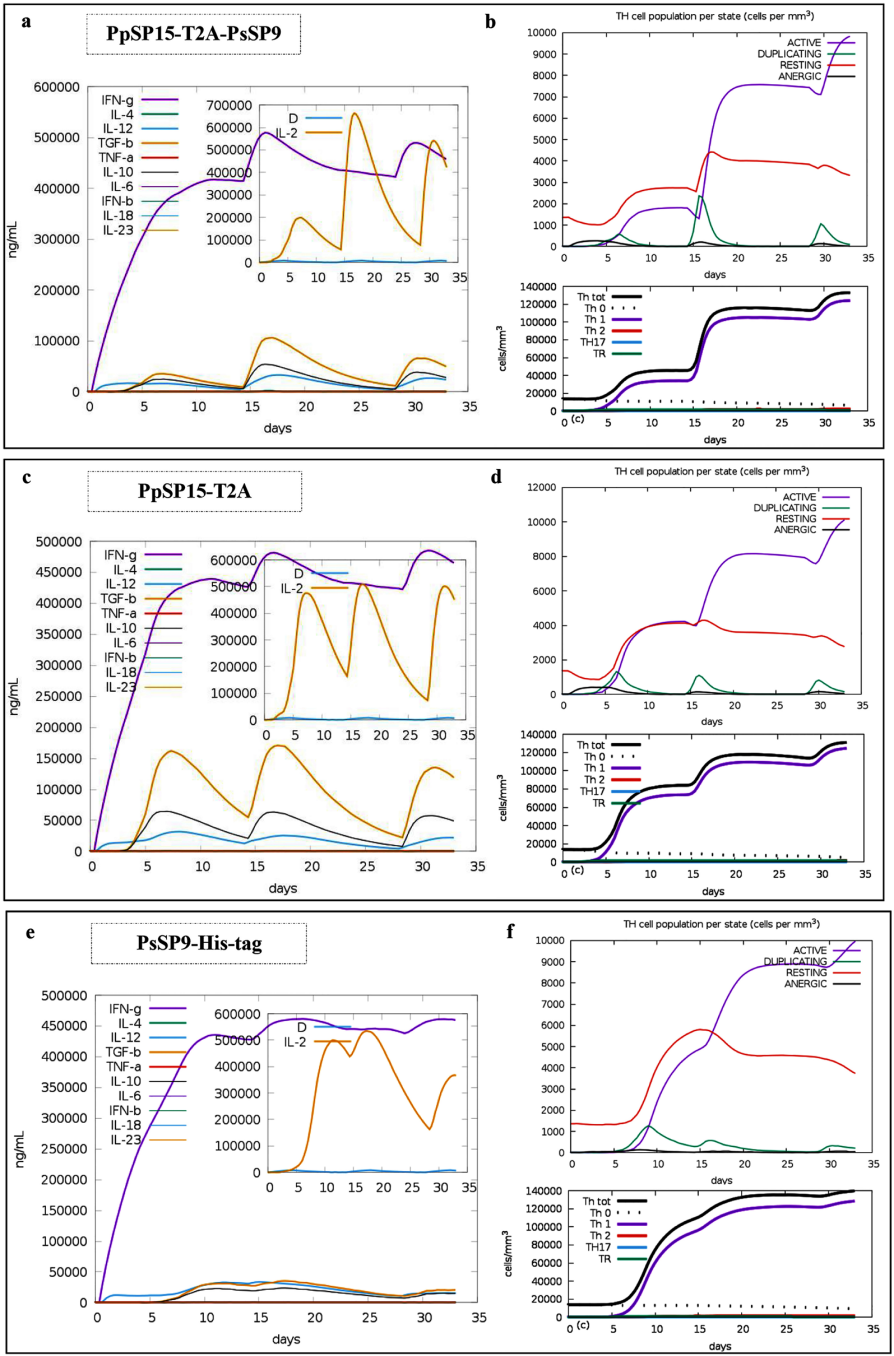
The outputs of immune simulation using the C-ImmSim server for the vaccine candidate construct. The immunogenicity of the vaccine candidate construct was evaluated using the C-ImmSim server after three injections at time steps 1, 42, and 84, with 2-week intervals. **a**, **c**, **e** Cytokine responses. The level of cytokines is illustrated in the main plot. In addition, the inset plot indicates the IL-2 level and the diversity index (D). The low value of the D index (blue line) indicates the high diversity of clones. **b**, **d**, **f** (upper panel) T helper cell populations. The resting state implies cells not presented with the antigen, while duplicating state indicates cells in the mitotic cycle. The anergic state shows the T-cell tolerance to the antigen. **b**, **d**, **f** (bottom panel) the differentiated T cell clones. Negligible Th2 and Th17 clones are detected

Different components of host defense, including innate and adaptive immunity elements, are involved in the successful clearance of the *Leishmania* parasite. The CD4^+^ Th1 cells are the key players in the host immune defense to mediate resistance and control over the *Leishmania* infection. These cells are among the important sources for producing IFN-γ and can activate and shift the macrophages into the M1 phenotype, resulting in NO production and eventually parasite elimination. Herein, the immune simulations of the PpSP15-T2A-PsSP9 vaccine candidate and its constituting proteins (PpSP15-T2A and PsSP9-His-tag) were evaluated in silico using the C-ImmSim online tool (Fig. 6). As indicated in Fig. 6a, considerable amounts of IL-12 (light blue) are produced in response to PpSP15-T2A-PsSP9, which results in the production of significant amounts of IFN-γ (purple). Likewise, the levels of IL-10 (black line) and TGF-β (orange line) as anti-inflammatory cytokines are much lower than IFN-γ. Therefore, these findings demonstrate a deviation of the immune response toward Th1, which is necessary to control the *Leishmania* infection course. Furthermore, in the inset plot of the same figure, a high level of IL-2 (orange line) particularly following the second dose of the PpSP15-T2A-PsSP9 vaccine candidate, was also evident which leads to T cell proliferation. Of note, the Simpson index (*D*) for clonal specificity investigation indicates possible diversity in immune responses induced by PpSP15-T2A-PsSP9. The cytokine profile and *D*-factor are reflected in the differentiation and diversity of CD4^+^ T cells population. As indicated in Fig. 6b, following the second administration of PpSP15-T2A-PsSP9, the level of active and duplicating T helper cells increases dramatically (Fig. 6b, top panel), which are mainly of the Th1 type (almost 90%) and not Th2 and Th17 (almost zero percent) (Fig. 6b, bottom panel). Of note, the dissociation components also produce high amounts of IFN-γ (Fig. 6c and e) after triple immunization (purple line). PpSP15-T2A induces high levels of TGF-β and IL-10 compared with PsSP9-His-tag, but remarkable levels of IFN-γ account for Th1 type CD4^+^ T cells differentiation. Similarly, both dissociation components induce Th1 differentiation rather than Th2 and Th17 (Fig. 6d and f bottom panel). Overall, it is postulated that PpSP15-T2A-PsSP9 as the selected vaccine candidate can activate Th1 immunity, which is highly critical in controlling the CL infection.

### Cloning and expression of the selected construct in *L. tarentolae*

**Fig. 7:**
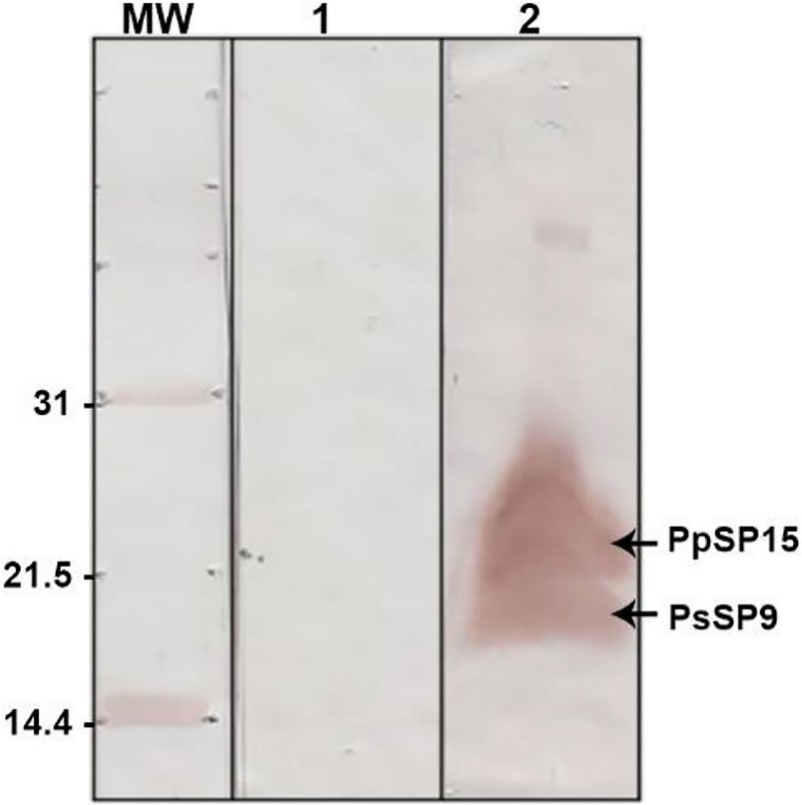
Protein expression confirmation in the supernatant of recombinant *L. tarentolae* PpSP15-T2A-PsSP9 using Western blot. Recombinant *L. tarentolae* parasites were cultured in M199 medium. Five days later, supernatants were collected and mixed with Ni-NTA agarose beads and loaded on 17.5% polyacrylamide gel. Lane 1, *L. tarentolae* control w/o insert, Lane 2, salivary proteins (PpSP15-T2A is about 18 kDa and PsSP9-His-tag is 15 kDa) detected in the supernatant of recombinant *L. tarentolae* (*L. tarentolae* PpSP15-T2A-PsSP9)

The main purpose of the present study was to select the best structurally valid combination of two different sand fly salivary proteins for further cloning in *L. tarentolae*. To reach this end, in silico cloning was performed in the pLEXSY-neo2 vector using the SnapGene tool (Additional file 1: Fig. S5) to assure the in-frame cloning of the PpSP15-T2A-PsSP9 sequence (Additional file 1: Fig. S6). The candidate sequence was then sub-cloned into the pLEXY-neo2 vector by *Bgl*II/*Nhe*I digestion and *L. tarentolae* parasites were further transfected with recombinant vectors. Western blot analysis of protein expression in stable recombinant *L. tarentolae* (*L. tarentolae* PpSP15-T2A-PsSP9) detected two separate bands about 18 kDa (PpSP15-his-tag-T2A) and 15 kDa (PsSP9-his-tag) in size indicating full dissociation of the fusion protein into constituent components in *L. tarentolae* (Fig. 7)*.* The immunogenicity and protective effect of this novel live vaccine candidate against *L. major*- and *L. tropica*-caused CL were evaluated in BALB/c mice model elsewhere [46].

## Discussion

CL is the most common form of leishmaniasis endemic in many countries around the world. One of the best control strategies is prophylactic vaccine development [1]. Unfortunately, despite many efforts, there is still no marketed human vaccine. Perhaps one of the main reasons for this failure is that most of the studies have focused only on parasite-related components [3]. Recently, sand fly-derived components such as saliva [10, 11] and midgut microflora [47] have also been shown to strongly affect immune responses against leishmaniasis. In recent years, several studies have examined the immunogenicity of salivary proteins from different species of sand flies [48, 49]. This has resulted in the identification of multiple saliva-related Th-1 stimulatory components as potential vaccine candidates [6] including proteins called PpSP15 from *Ph. papatasi* [9] and PsSP9 from *Ph. sergenti* [50]. Noteworthy, an effective prophylactic vaccine might benefit a combination of all parasite- and vector-related factors. In line with this concept, in our previous study [45], the immunogenicity of *L. tarentolae* expressing cysteine proteinases (CPA-CPB) along with PpSP15 DNA was assessed against *L. major* and a higher protective immunity was obtained in comparison with recombinant *L. tarentolae* alone. Since the effect of sand fly salivary proteins as vaccine candidates could be species-specific, we aimed at designing a saliva-based *L. tarentolae* dual vaccine candidate targeting two main pathogenic *Leishmania* species in the Old World, *L. major* and *L. tropica*, using two different salivary proteins, PpSP15 and PsSP9, and a detailed in silico mRNA and protein structure analysis. Of note, this approach could be particularly relevant in endemic regions with various sand fly vectors.

Over the past few decades, it has been shown that the use of multiple-antigen protein fusion in the development of multivalent vaccines is useful for stimulating a stronger immune response [51]. In this regard, Cecílio et al. introduced a fusion protein consisting of two immunogenic salivary proteins, PdSP15 and LJL143, as potential vaccine candidates against CL and VL [52]. In this fusion protein, based on epitope mapping, two immunogenic parts of each protein were placed in the final sequence without using linkers [52]. Regarding fusion proteins, misfolding and aggregation of proteins are considered the two main challenges. Proper folding and structural stability of a protein increases its processing and presentation by APCs [53]. Moreover, the creation of immunodominant junctional epitopes can lead to immune response deviation [54]. To solve these problems, the use of linkers between proteins is recommended [55]. Linkers fall within different categories (based on their structure and function) including cleavable peptides. In this study, a cleavable 2A peptide (T2A) was used between two salivary proteins. *Thosea asigna* virus-derived self-cleavage 2A peptides are 18–22 amino acids that, by the ribosome-skipping mechanism, cause cleavage in their C-terminal region and eventually lead to the separate production of flanking proteins in almost equal concentrations concurrent with protein synthesis [56]. This was of paramount importance in our study, since we aimed at designing a multicomponent candidate vaccine targeting both *L. major* and *L. tropica*, and the equal production of both vaccine candidates was crucial in APCs. Linker insertion can further add to the complexity of fusion proteins in structure and folding. This necessitates preliminary review of all possible combinations when two or more proteins are to be fused together [57]. In other words, all possible combinations must be evaluated at the RNA and protein levels to find the most favored fusion with respect to mRNA secondary structure and protein folding. The possible combinations in this study included PpSP15-T2A-PsSP9, PsSP9-T2A-PpSP15, PpSP15–PsSP9, and PsSP9–PpSP15.

The secondary structure of mRNA is a key element impacting gene expression [58]. For many years, the prediction of RNA secondary structure was based on the MFE of the optimal structure, but later, a new concept called “centroid structure” was introduced for more accurate prediction [59]. Studies have shown that a “centroid structure” versus an “optimal structure” was able to reduce prediction error by 30% [19]. Therefore, in this study, the prediction of mRNA secondary structure was carried out using the RNAfold server, which relies on various criteria, including the MFE of optimal structure, MFE of centroid structure, ensemble diversity, and free energy of the thermodynamic ensemble. Previously, we needed to estimate the full-size mRNA sequence transcribed in *L. tarentolae*. In *Leishmania* parasites, such as other trypanosomatids, genes are transcribed as polycistronic units, and mature mRNA for each gene is obtained through trans-splicing and polyadenylation events in the intergenic regions [60]. There are no strict rules to indicate the exact splice acceptor sites, and variations occur from gene to gene [60]. Nevertheless, a comprehensive literature review indicated that the trans-splice acceptor site is the first “AG” dinucleotide after the longest polypyrimidine tract found at the 5′ (and 3′) Utr flanking the genes [61, 62]. The polyadenylation site is further estimated to fall within 500 nucleotides upstream of the 3′ trans-splice acceptor site. Unlike other eukaryotes, there are no distinguished signals for polyadenylation events in *Leishmania,* and the length of poly-A tails varies largely from gene to gene [63]. Here, we compared the short (12 adenine), intermediate (50 adenine), and long (100 adenine) tails with respect to the RNA secondary structure and found no remarkable difference among them (data not shown). When the full-size mRNA sequence which is likely to be produced in the *Leishmania* parasite was completed, we further analyzed the sequence of all four different combinations with RNAfold. The results indicated that the PpSP15-T2A-PsSP9 mRNA is more stable than any other combination by the lowest free energy of the optimal and centroid structures and the lowest ensemble diversity (an indicator of stable structure).

The RNA structure is the key factor controlling the protein production level. Structural analysis of possible mRNAs gave us a strong clue that the 5′PpSP15-3′PsSP9 linked with the T2A peptide generates the most stable secondary structure among the rest of the combinations, and the individual proteins will be further produced from this RNA. Keeping in mind that the cleavage efficiency of 2A peptides during protein synthesis is not 100% and that full-sized protein could be produced besides individual components due to occasional unsuccessful ribosome skipping [62], we proceeded through protein structure analysis besides mRNA. Moreover, after cleavage, the T2A peptide remains linked to the N-terminal protein, impacting the final folding. Therefore, it was necessary to analyze all possible combinations using homology modeling for further confirmation. Homology modeling is a bioinformatics approach used to predict protein structure based on its amino acid sequence. It was developed to predict the function of an unknown protein based on the homology of its tertiary structure with similar proteins [63]. Here, we used this approach to select between combinations based on the predicted tertiary structures assuming that the more accurate 3D model is more likely to be expressed in biological systems. For this, all possible combinations were modeled using I-TASSER, and after refinement, they were evaluated using structural validation tools. Although most predicted 3D structures were found to be valid by different validation approaches, the QMEAN results showed slight differences at the local stability level. Generally, the combinations of 5′-PpSP15 and 3′-PsSP9 had higher local stability than the others. However, the T2A region stability and its downstream signal peptide slightly decreased in PpSP15-T2A-PsSP9, which could be due to the binding of these regions to each other. For this, the PpSP15-T2A-PsSP9 combination without a signal peptide for the downstream gene (PsSP9) was also evaluated (data not shown). The findings indicated an increase in the stability level of the T2A region. As a result, the downstream signal peptide could influence the structural stability. Using the signal peptide for the downstream gene is a controversial issue in T2A linker insertion. Some people believe that the T2A downstream protein could enter the endoplasmic reticulum system without the signal peptide through the slip streaming mechanism. Others believe that this mechanism does not exist in eukaryotes and that signal peptide usage for downstream protein secretory expression is still required. Further in vitro protein production assessment is required to confirm the equivalent release of both components.

To further confirm the proper folding, a 3D model of all combinations was overlaid on the predicted model of PpSP15, PsSP9, and the PDB structure of PdSP15. This was to determine the level of resemblance between the predicted model and each individual original protein. According to the results, all constructs were very well superimposed, and their structural stability was verified. Since PdSP15 was previously modeled and confirmed using crystallographic analysis, superimposing results with this protein was important. This result indicated that the homology modeling approach applied in this study almost accurately predicted the most likely model. As a result, the 5′-end PpSP15 and 3′-end PsSP9 arrangement was preferred, and PpSP15-T2A-PsSP9 was selected in line with the RNA prediction results. The AlphaFold2 server was used in parallel to validate the candidate construct selected through homology modeling. The whole PpSP15-T2A-PsSP9 protein and cleavage components indicated acceptable structural criteria, with T2A potentially agitating the local pLDDT and PAE also predicted by the QMEAN local quality score. Therefore, this newly developed protein structural analysis tool can be used in a homology modeling approach for precise decision-making in multi-protein design.

## Conclusion

The current study provides a detailed in silico strategy for designing fusion proteins in *L. tarentolae* using a cleavable linker. Epitope mapping results indicated that this linker is not immunogenic and generates no neoepitopes when inserted between the two proteins. The PpSP15-T2A-PsSP9 activates appropriate immune responses through IFN-γ production by CD4^+^ Th1 cells, which is predicted in silico here and is evaluated in vivo elsewhere [46]. According to the population coverage results, this candidate vaccine could be effective in the Old World, especially for the regions with the highest numbers of CL cases.

## Supplementary Information

**Additional file 1: Table S1.** Nucleic acid and protein sequences of all components needed for construct design. **Figure S1.** Complete sequence of Utr1 and Utr2 of LEXSYcon2 vector. Trans-splicing sites used in mRNA synthesis are highlighted in blue (longest polypyrimidine tract), red (splice acceptor site), and purple (polyA site). Utr1 and Utr2 sequences are obtained from the LEXSYcon2 Expression Kit’s manual (Jena Bioscience). **Figure S2.** Optimal and centroid secondary structure analysis by RNAfold for all mRNA combinations. The full-sized mRNA sequence of all combinations was submitted to the RNAfold web server for secondary structure prediction. This server predicts two secondary structures, including optimal and centroid structures. a-column: Optimal secondary structure. b-column: Centroid structure. **Table S2.** Characteristics of Galaxy refined models. **Figure S3.** Superimposition of all combinations with the original proteins. The validated 3D model of each combination was compared with the original proteins, PpSP15, PsSP9, and PdSP15, using UCSF Chimera v1.15 software. The Superimposition was applied to indicate similarity between two overlaid protein structures by calculating RMSD. For closely homologous proteins, the RMSD is as small as 3 Å. The 3D model color: copper for PpSp15-T2A-PsSp9, PpSp15–PsSp9, PpSP15-Histag-T2A, and PpSP15-Histag. Yellow for PsSP9-T2A-PpSP15, PsSP9–PpSP15, and PsSP9-His-tag-T2A. Red for PpSp15, PsSP9, and PdSP15. **Table S3.** High-scored HLA-II junctional epitope. **Table S4.** High scored helper and CTL epitopes of vaccine constituent proteins. **Figure S4.** Estimation of population coverage of PpSP15 + PsSP9 combined vaccine in the Old World. Population coverage of the predicted epitopes was evaluated with the IEDB population coverage tool. **Table S5.** Physicochemical characteristics of the vaccine construct compared with original PpSP15 and PsSP9 components. **Table S6.** Antigenicity and allergenicity of the candidate vaccine construct compared with the original PpSP15 and PsSP9 components. **Figure S5.** In silico cloning of the vaccine candidate into the pLEXSY expression vector using SnapGene software. The red part shows the PpSP15-T2A-PsSP9 target sequence between *Bgl*II and *Nhe*I restriction sites. **Figure S6.** In frame cloning of PpSP15-T2A-PsSP9 construct into the pLEXSY expression vector by SnapGene software.

## Data Availability

Supporting data for the conclusions of this article are included within the article and its additional files. The raw datasets used and analyzed in this study are available upon reasonable request.

## Abbreviations

CL: Cutaneous leishmaniasis
MCL: Mucocutaneous leishmaniasis
VL: Visceral leishmaniasis
*L.*: *Leishmania*
DTH: Delayed-type hypersensitivity response
*Ph*.: *Phlebotomus*
T2A: *Thosea asigna* virus 2A peptide
Utr: Untranslated region
SL: Splice leader
MFE: Minimum free energy
3D: Three-dimensional
RMSD: Root-mean-square deviation
HTL: Helper T lymphocyte
ANNs: Artificial neural networks
KNN: *K*-nearest neighbors
His-tag: Histidine-tagged
*C*-score: Confidence score
APCs: Antigen-presenting cells
pLDDT: Predicted Local Distance Difference Test
PAE: Predicted Aligned Error
