# Supplementary material for: Structural analysis of PpSP15 and PsSP9 sand fly salivary proteins designed with a self-cleavable linker as a live vaccine candidate against cutaneous leishmaniasis
Source: Parasit Vectors. 2022 Oct 19;15:377. doi: 10.1186/s13071-022-05437-x (PMC9580450; doi:10.1186/s13071-022-05437-x)

| Components | Nucleotide sequence | Protein sequence |
| --- | --- | --- |
| *Bgl*II | AGATCT | - |
| Kozak sequence | ACC | - |
| *Leishmania* signal peptide sequence | ATGGCCTCGAGGCTCGTCCGTGTGCTGGCCGCCGCCATGCTGGTTGCAGCGGCCGTGTCGGTCGACGCTGGCGCCTCTCTAGAC | MASRLVRVLAAAMLVAAAVSVDAGASLD |
| PpSP15  (codon optimized) | GAAAACCCATCTAAGAAATGCGAAGAGAAGTTCAAAAACGACGCCTCCAAAATGGCTTGTATCCCCCACTGCAAATACCAGTATTACGGGTTTGTTGCTATGGACAACAACATCGCCCGTCCCGAGATTCGTAAATTTTCTGATGTGCTTATCAAGTACAACGTGGTTGATAAAAGCCTTAAGGCTGACATCCGTAAAATCATGCATGAGTGCGCCAAAAAGGTCAAAAAACAAGCTCGGGAGGATTCGCATTGGCTCAACTGTCGCACAACAATTAACTATTATCGGTGTATCCTGACTGATAAGCGTATCGGTCCACAACGCTTTGACCGCGCAATCGAGGAATATGACAAAACAATCAACATA | ENPSKKCEEKFKNDASKMACIPHCKYQYYGFVAMDNNIARPEIRKFSDVLIKYNVVDKSLKADIRKIMHECAKKVKKQAREDSHWLNCRTTINYYRCILTDKRIGPQRFDRAIEEYDKTINI |
| His-tag | CACCATCACCACCACCAC | HHHHHH |
| Linker | GGAAGCGGA | GSG |
| T2A (JA365321.1\| Thosea asigna virus) | GAGGGCAGGGGAAGTCTTCTAACATGCGGGGACGTGGAGGAAAATCCCGGCCCC | EGRGSLLTCGDVEENPGP |
| PsSP9  (codon optimized) | GGCAATCCATCCAAAAAGTGTCGTGAAGACTACCGGGCAAAAAAACTGGACGAATCTTGTATCCTCCATTGTGAATACCGTGCTTACGGGTTTTCCAACGACAAATATGACATTAAGAAAAAGCAAATCGACAAATTTGTTGAAGTGCTGATTAACGCAAAAGTGGTTGACAGCTCTGATCGGACGAAGCTGGACAATCTTCTCCGCAAATGTGCTAACCAGGCCCGCTCCAAGCACTCGAACAAACTCAATTGCTACACAACAATCGATTATTACCGGTGTGTTGTGAATGATGAATCTCTTATCAATTATCGTAAATTCGTTGGCGCAATCATGGCGTATGACAAGACAATCAACATC | GNPSKKCREDYRAKKLDESCILHCEYRAYGFSNDKYDIKKKQIDKFVEVLINAKVVDSSDRTKLDNLLRKCANQARSKHSNKLNCYTTIDYYRCVVNDESLINYRKFVGAIMAYDKTINI |
| Stop codon | TGA | * |
| *Nhe*I | GCTAGC | - |

**Table S1. Nucleic acid and protein sequences of all components needed for construct design**

**Fig S1. Complete sequence of Utr1 and Utr2 of LEXSYcon2 vector.**

Trans-splicing sites used in mRNA synthetize are highlighted in blue (longest polypyrimidine tract), red (splice acceptor site) and purple (polyA site). Utr1 and Utr2 sequences are obtained from the LEXSYcon2 Expression Kit’s manual (Jena Bioscience).

**Utr1:** Non-translated region of *aprt* gene of *Leishmania* with splice acceptor site for target gene

AGTCGCAGCCTGACCGCATCACACATCAAGGCGTTACAGCCTCTCCGTCTTTGCTGCACCCAAGTGTATTCGTGTGACCGCGTTGAGGGATCTAATGGATTCGGAACTTTGGTCGTTTGGTCTCTCATGCTGCCCAATCCCATGCGCTTTTCTCCGTGCCTCTCTGTCTCCCCTTCCGTTCTATTTGGACAACGTGTACTGTAATGCGTGCGCCATCTGACAACGAAT**AG**ATCAGCAGCATTCGCACACTTGCACATACCCAGTGAAGCTTTTGTGTCTGTCGTATTGACAACACCGACTGCAACAAGGTGTAGATAGAAGTTGGCCTTCTCGCTCGCTCGCACGCTCTTCACGCTCCTGCTTTCCTTGCTGTGCCTTGCCACC

**Utr2:** 1.4k intergenic region from *cam* operon of *Leishmania* with polyA site for target gene and splice acceptor site for marker gene

CTCCTCCTCCTTTCTTGTTCCTTTCACGTCGCCTTCTCGGTTGTAGCTGGCAGACGACGAGTCTTACTTTTACGTGTACTTCTCTATAGATGATGTATGATCTCTCTGCATGCGTGTTCGTGCATGTGTCCGTGTGTTGTG**TA**CGCGTGCGTCTCGCCTCAGCTCTCCGCGTGAAAGGGTTTGACTGCCCATGATGCGTGTGTATATCCACGCGCAGGCACGCACACACACACACACACACACACACACAGGCACACACAGGCACACAAACGCATCTCAGGCCGAGCCGCATACGTCTCTCGCACGGTCTCGTTTATTTGATCATGTAGTTGAGTATTAAATTGGGAAGACAAAAACATAATAGCACGAAGAGTCGGGCACGAAAAGCCCGATCTCTCTCTCTCTCTCTCGTGCGCGCAGGGGCGTGTGGGTGCACGACGACGAGGACGGAGGGGGGAAGGGAGGCATAAACGGATCGATCCCCCATCGCACATGCGGGTACGAGCATTATCTGCTGTGTCTGGTCCTTTATCATATCGCTACCCGCCCGCCCCCCGCCCCCCTCCCCCCCCCCCCCCCGCGGCTCCTGTCACTGTCGTTCCTGCGACTCCCCACACACCCGCGCAACGCTCATGTCACGAAGAGAAAAGTAGAAGGCGTGGCGATGCGTTGCGCGGCCCCCTGTCCGCTGGCATGCAGACGTCGAGCCGTGGAACGGATGGTAGCGAGAGACAACGCGGCGATGCAGGAAAGGAGATTTACTGCAGGACGTCTACACACGCGCGCACACCACTGCGAATGGCACCGTGCTGAGGAAAATTGGGGGGGAGGGCGCACGGGGGCGGGGCAGGAAACGGTTGGATCAGCAGCACTCTCAACTTGTGTCCGTATTGCGAAGGAGGGAGAGGAGCCTGCCCGTATTATGGGCGCTGAAACGTCGGAACCACCTATGAATCCCACTTCTGCGTCGCGGAGGCGTACTGCTTCCGACCACCTCCGCAGCTCACGCGCGTCGAGCTCCCTCCCTTTCCCCTTCTTTCCGTTGTGTGTGTGTGTGTGTGTGTGTCTTGTCACGATGCATGGCCATCTCTCCTCCAACCCTCCACGCCTTCCTCTCCCCCGCCCATCAAACGCGCTACGGCCACAACCATTTCTTCAAGTATCACATCCACCACCACTCTTACCCACCTACCCTCTTCGACTCTGACACAGCCACTGACGCCCTCTCCGCCTCTCTCGCTCGCTGCACATTGACTCTCCACAGCCCCCCCTCCCTCGCACAGCCAGCCCCACCCACCCACCCACCCACCCACCGCTCTACACACCTCCACAGCAGCC

**utr2**

**utr1**

**pLEXSY vector**

**Target gene**

**Marker gene**

**Fig S2**. **Optimum and Centroid secondary structure analysis by RNAfold for all mRNA combinations.**

The full-sized mRNA sequence of all combinations was submitted to the RNAfold web server for secondary structure prediction. This server predicts two secondary structures including optimum and centroid structures. **a-column**: Optimum secondary structure. **b-column**: Centroid structure.


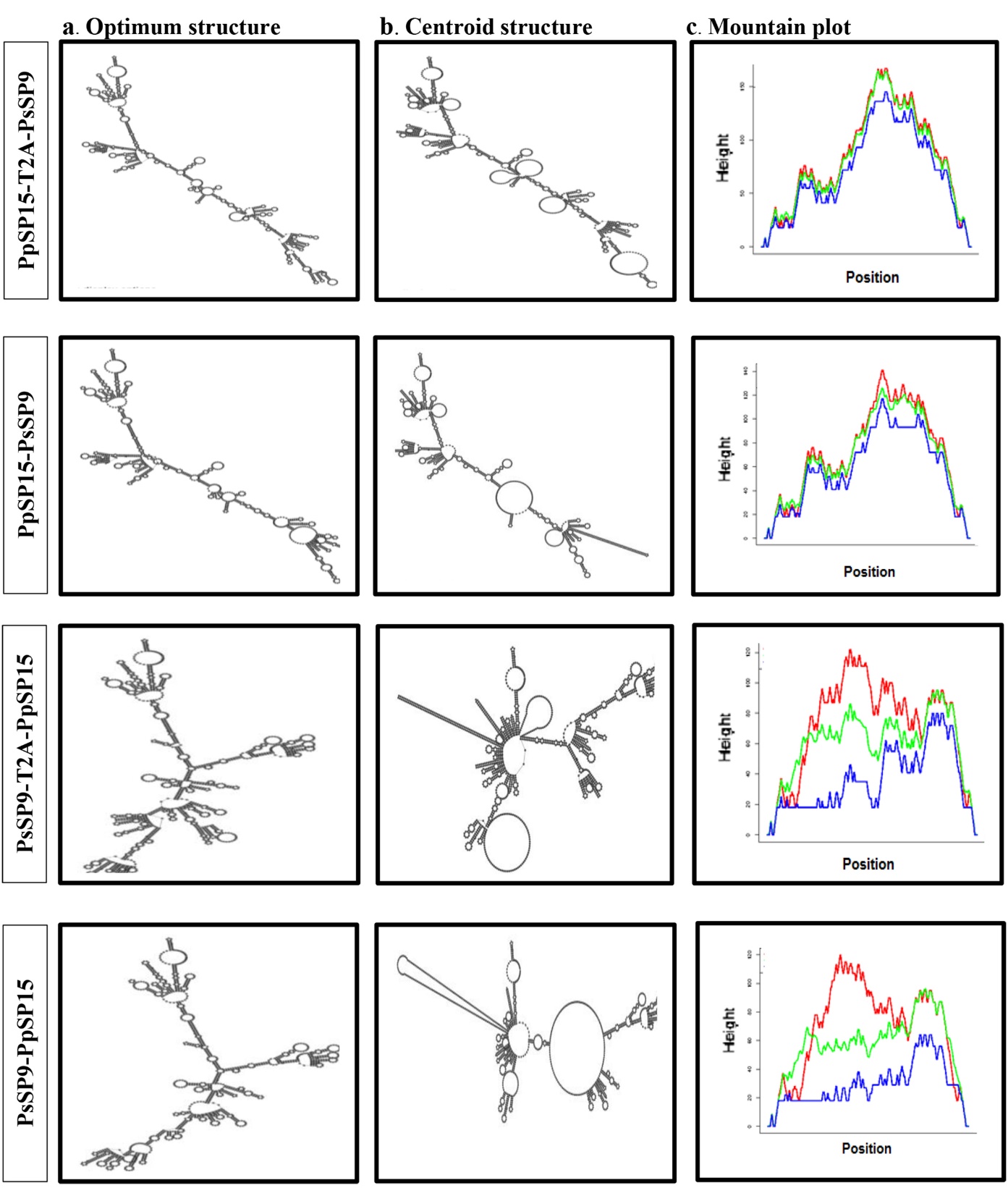


**Table S2. Characteristics of Galaxy refined models**

| **Constructs** | **^1^c-score** | **^2^GDT-HA** | **^3^RMSD** | **^4^Rama favored** |
| --- | --- | --- | --- | --- |
| **PpSP15-T2A-PsSP9** | -2.49 | 0.9109 | 0.545 | 84.5 |
| **PsSP9-T2A-PpSP15** | -2.65 | 0.8521 | 0.649 | 79.3 |
| **PpSP15-T2A** | -0.10 | 0.9561 | 0.410 | 91.8 |
| **PsSP9-T2A** | -0.30 | 0.9606 | 0.385 | 91.7 |
| **PpSP15-His-tag** | 1.11 | 0.9902 | 0.272 | 98.4 |
| **PsSP9-His-tag** | 1.01 | 0.9802 | 0.390 | 98.4 |
| **PsSP9-PpSP15** | -0.56 | 0.9435 | 0.425 | 86.2 |
| **PpSP15-PsSP9** | -0.12 | 0.9405 | 0.438 | 91.5 |
| **PpSP15** | 1.50 | 0.9898 | 0.284 | 95.9 |
| **PsSP9** | 1.60 | 0.9896 | 0.306 | 96.6 |

^1^ is a confidence score for estimating the quality of predicted models by I-TASSER

^2^ is a meter of similarity between two [protein structures](https://en.wikipedia.org/wiki/Protein_structure)

^3^ is an average distance of all residue pairs in two structures

^4^ residues in most favored regions

**Fig S3. Superimposition of all combinations with original proteins**

The validated 3D model of each combination was compared to original proteins, PpSP15, PsSP9, and PdSP15, using UCSF Chimera v1.15 software. The Superimposition was applied to indicate similarity between two overlaid protein structures by calculating RMSD. For closely homologous proteins, the RMSD is as small as 3 angstrom. The 3D model color: copper for PpSp15-T2A-PsSp9, PpSp15-PsSp9, PpSP15-Histag-T2A, and PpSP15-Histag. Yellow for PsSP9-T2A-PpSP15, PsSP9-PpSP15, and PsSP9-His-tag-T2A. Red for PpSp15, PsSP9, and PdSP15.

**PdSP15**

**PpSP9**

**PpSP15**

**
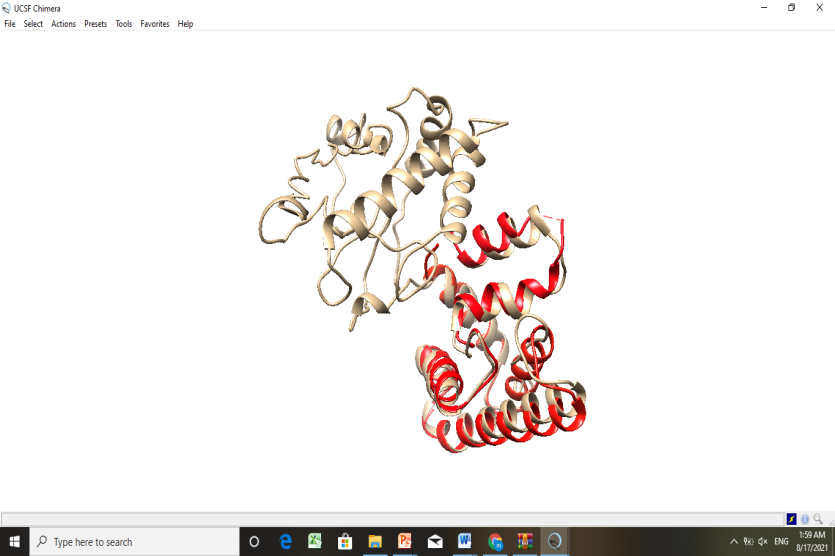

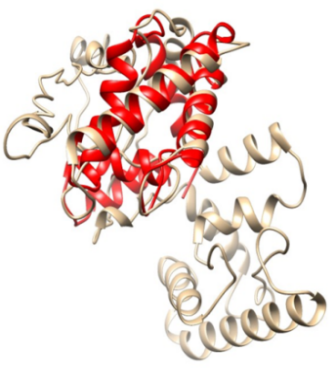

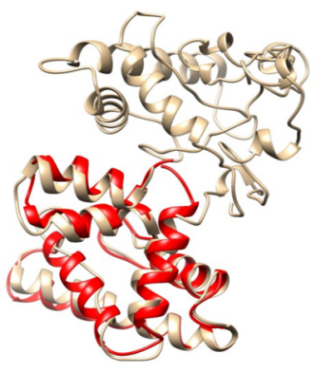
**

**PpSp15-T2A-PsSP9**

**
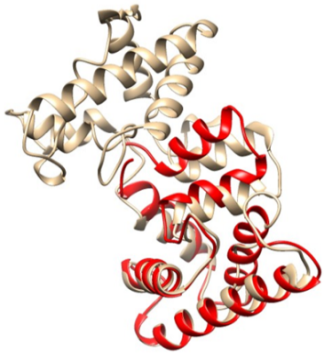

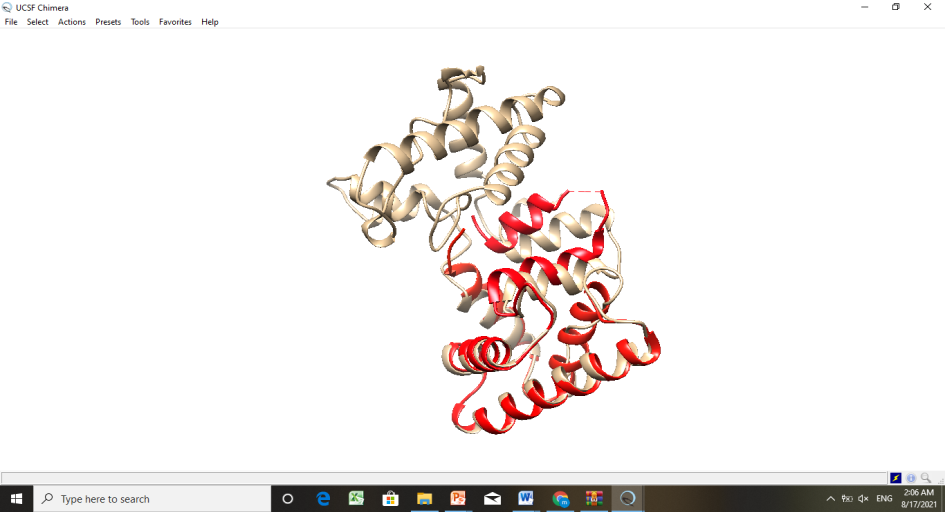

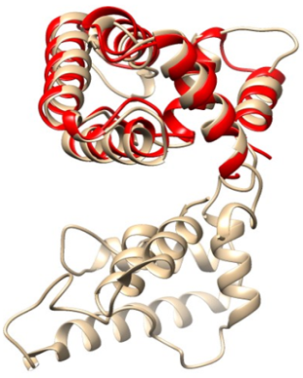
**

**PpSp15-PsSP9**

**
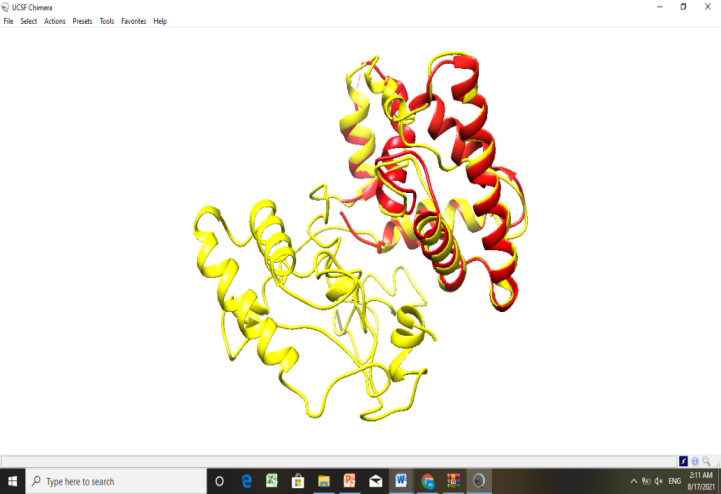

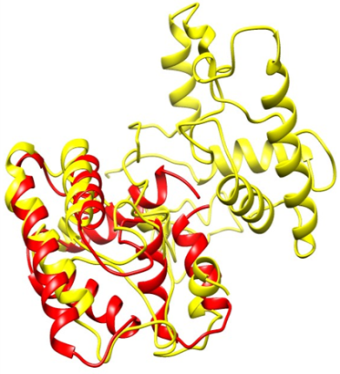

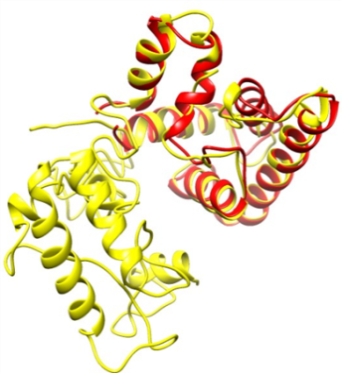
**

**PsSP9-T2A-PpSP15**

**
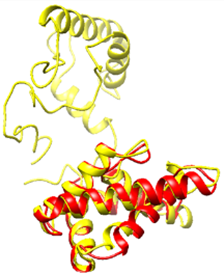
**
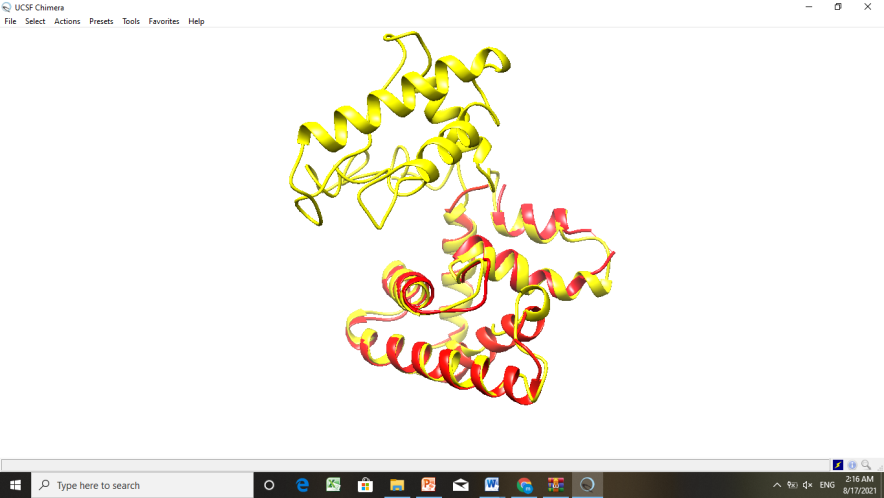
**
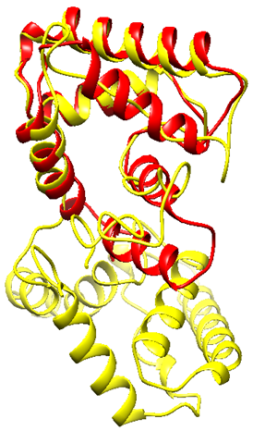
**

**PsSP9-PpSP15**

**PdSP15**

**PpSP9**

**PpSP15**


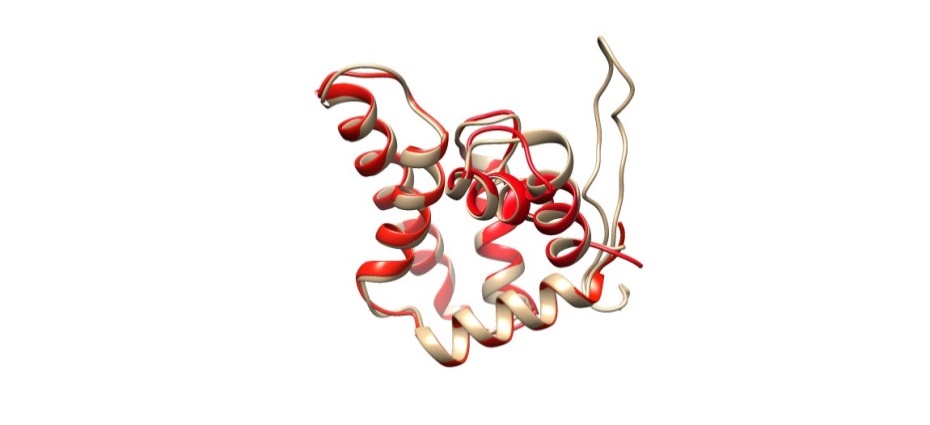

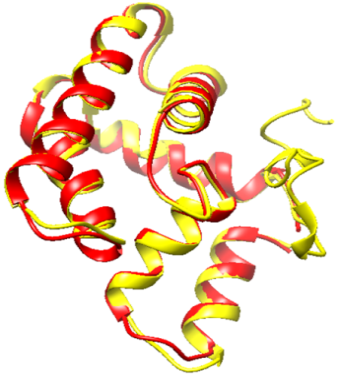


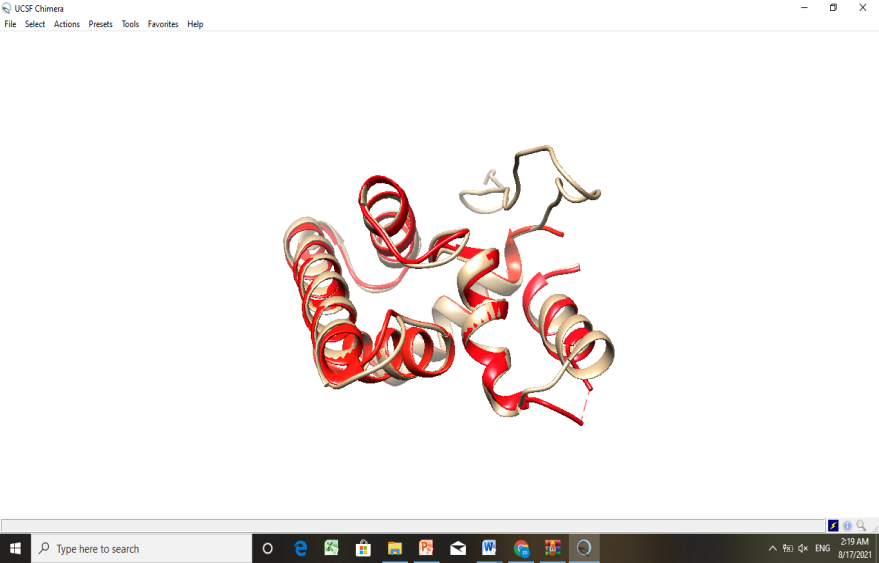


**PpSP15 -T2A**

**PpSP15-T2A**

**PsSP9-T2A**

**PpSP15-His-tag**


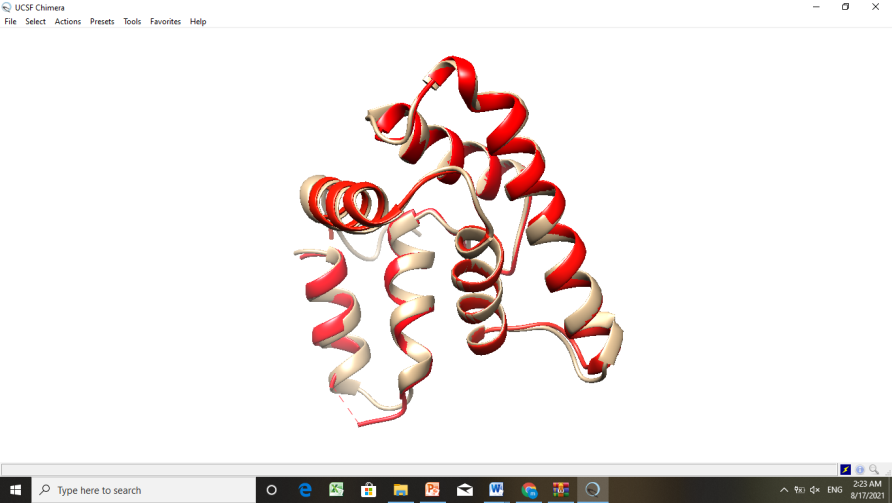

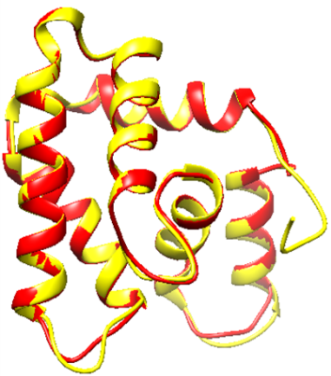

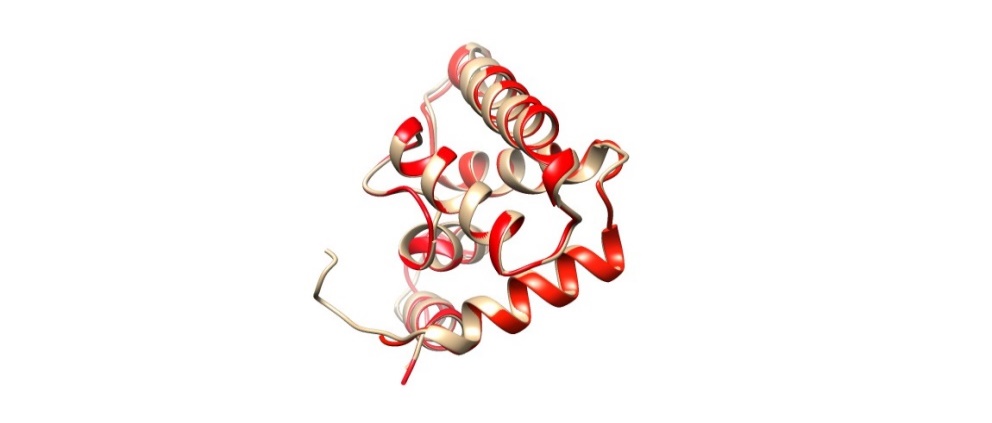


**PsSP9-Histag**

**PpSP15-Histag**

**Table S3. High scored HLA-II junctional epitope**

| **HTL epitopes** | **Peptides** | **HLA Allele** | **Score** | | | |
| --- | --- | --- | --- | --- | --- | --- |
|  |  |  | **IEDB** | **SYFPEITHI** | **NetMHCIIpan** | **RANKPEP** |
| **PsSP9-PpSP15** | GAIMAYDKTINIENP | HLA-DRB1*03:01 | - | 22 | 0.852386 | - |
|  | GAIMAYDKTINIENP | HLA-DRB1*04:01 | - | 26 | 0.664034 | - |
|  | VGAIMAYDKTINIEN | HLA-DRB1*03:01 | 3.4 | - | 0.793643 | - |
|  | IMAYDKTINIENPSK | HLA-DRB1*07:01 | - | 24 | - | 12.327 |

**Table S4. High scored helper and CTL epitopes of vaccine constituent proteins**

| **Score** | | | | | **^1^HLA Allele** | **Peptides** | **HTL**  **epitopes** |
| --- | --- | --- | --- | --- | --- | --- | --- |
| **^6^IFNepitope** | **^5^RANKPEP** | **^4^NetMHCIIpan** | **^3^SYFPEITHI** | **^2^IEDB** |  |  |  |
| 0.43257921 | 5.414 | 0.66 | 22 | 0.26 | HLA-DRB1*04:01 | KYQYYGFVAMDNNIA | **PpSP15-T2A** |
| 0.45950706 | - | 0.03 | 22 | 0.24 | HLA-DRB1*04:01 | YQYYGFVAMDNNIAR |  |
| 0.43765147 | 15.604 | 0 | 22 | 0.26 | HLA-DRB1*04:01 | YYGFVAMDNNIARPE |  |
| 0.43765147 | 16.801 | 0.11 | - | 0.16 | HLA-DRB1*04:05 |  |  |
| 0.45813766 | - | 0.01 | 20 | 0.26 | HLA-DRB1*04:01 | YGFVAMDNNIARPEI |  |
| 0.51414757 | 18.665 | 0.4 | 29 | 3.9 | HLA-DRB1*03:01 | YRCILTDKRIGPQRF |  |
| 0.43759942 | - | 0.58 | 27 | 3.2 | HLA-DRB1*03:01 | DKSLKADIRKIMHEC |  |
| 0.37880718 | - | 0.33 | 28 | 2.2 | HLA-DRB1*04:01 | EEKFKNDASKMACIP |  |
| 0.37081978 | 7.567 | 0.59 | - | 2.2 | HLA-DRB1*04:01 | CEEKFKNDASKMACI |  |
| 0.45239465 | - | 0.44 | 22 | 3 | HLA-DRB1*03:01 | GAIMAYDKTINIHHH | **PsSP9-His-tag** |
| 0.44966444 | 8.253 | - | 28 | 3.5 | HLA-DRB1*04:01 | IDYYRCVVNDESLIN |  |

| **CTL epitopes** | **Peptides** | **HLA-Allele** | **Score** | | | |
| --- | --- | --- | --- | --- | --- | --- |
|  |  |  | **^7^IEDB** | **^8^NetCTL** | **^3^SYFPEITHI** | **^5^RANKPEP** |
| **PpSP15-T2A** | KMACIPHCK | HLA-A*03:01 | 26.1 | 1.428 | - | 18.244 |
|  | KIMHECAKK | HLA-A*03:01 | 66.8 | 1.3293 | 24 | 21.333 |
|  | IMHECAKKV | HLA-A*02:01 | 282.2 | - | 23 | 64 |
|  | CRTTINYYR | HLA-B*27:05 | 296.8 | 0.8662 | 23 | - |
|  | HWLNCRTTI | HLA-A*24:02 | 252.8 | 1.1801 | - | 18.087 |
| **PsSP9-His-tag** | SLINYRKFVG | HLA-A*02:01 | - | 0.8796 | 24 | 77 |
|  | AIMAYDKTI | HLA-A*02:01 | - | 0.8117 | 21 | 79 |
|  | MAYDKTINI | HLA-A*02:01 | 462.7 | 0.9827 | - | 64 |
|  | NYRKFVGAI | HLA-A*24:02 | - | 1.1999 | 21 | 17.903 |
|  | RAYGFSNDKY | HLA-A*03:01 | 64.4 | 1.2892 | 20 | - |

^1^The frequent common allele in different servers

^2^ Threshold value: Percentile rank < 5%

^3^ Threshold value: Scores above 20

^4^ Threshold value: Percentile rank < 1% (strong binder)

^5^ Threshold value: PSBT of consensus binding epitope

^6^ Threshold value: <0.5 as positive

^7^ Threshold value: IC_50_ values less than 500 nM

^8^ Threshold value: above 0.75

**Fig S4**. Estimation of Population coverage of PpSP15+PsSP9 combined vaccine in the old world. Population coverage of the predicted epitopes was evaluated with the IEDB population coverage tool.

**Table S5. Physicochemical characteristics of the vaccine construct compared to original PpSP15 and PsSP9 components**

| **PsSP9** | **PpSP15** | **PsSP9-His-tag** | **PpSP15-T2A** | **PpSP15-T2A-PsSP9** |  |
| --- | --- | --- | --- | --- | --- |
| 14046.08 | 14526.82 | 14868.93 | 17265.67 | 35170.17 | **Molecular weight** |
| 9.19 | 9.30 | 9.19 | 8.92 | 9.12 | **Isoelecteric PI** |
| 13410 | 15930 | 13410 | 15930 | 29340 | **Extinction coefficient** |
| 24.30 (Stable) | 52.20 (Unstable) | 24.59  (Stable) | 45.84 (Unstable) | 33.64  (Stable) | **Instability index** |
| 78.00 | 69.59 | 74.29 | 64.59 | 74.61 | **Aliphatic index** |
| -0.736 | -0.835 | -0.853 | -0.913 | -0.682 | **Grand average of hydropathicity (GRAVY)** |

**Table S6. Antigenicity and allergenicity of candidate vaccine construct compared to original PpSP15 and PsSP9 components**

| **AllerTOP** | **ANTIGENpro** | **^1^VaxiJen** |  |
| --- | --- | --- | --- |
| NON-ALLERGEN | 0.814395 | 0.6904 (ANTIGEN) | **PpSP15-T2A-PsSP9** |
| NON-ALLERGEN | 0.814395 | 0.6327 (ANTIGEN) | **PpSP15-T2A** |
| NON-ALLERGEN | 0.672642 | 0.7240 (ANTIGEN) | **PsSP9- His-tag** |
| ALLERGEN | 0.494774 | 0.4778 (ANTIGEN) | **PpSP15** |
| NON-ALLERGEN | 0.693483 | 0.7074 (ANTIGEN) | **PsSP9** |

^1^Threshold value was set at 0.4

**Fig S5.** In silico cloning of the vaccine candidate into the pLEXSY expression vector using SnapGene software. The red part shows the PpSP15-T2A-PsSP9 target sequence between *Bgl*II and *Nhe*I restriction sites.


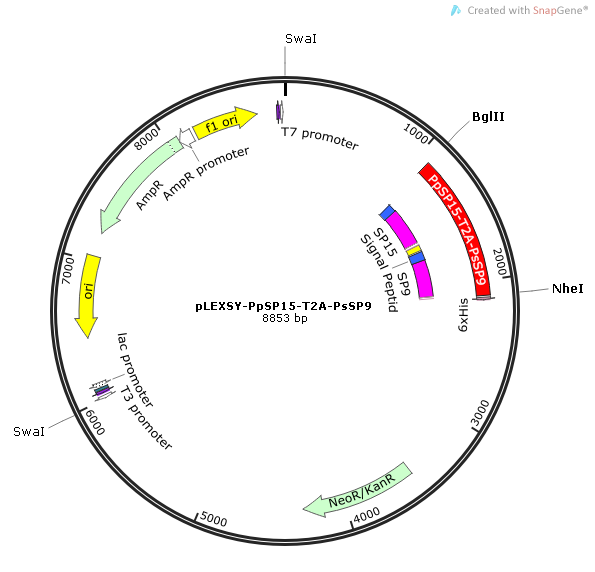


**Fig S6. In frame cloning of PpSP15-T2A-PsSP9 construct into the pLEXSY expression vector by SnapGene software.**


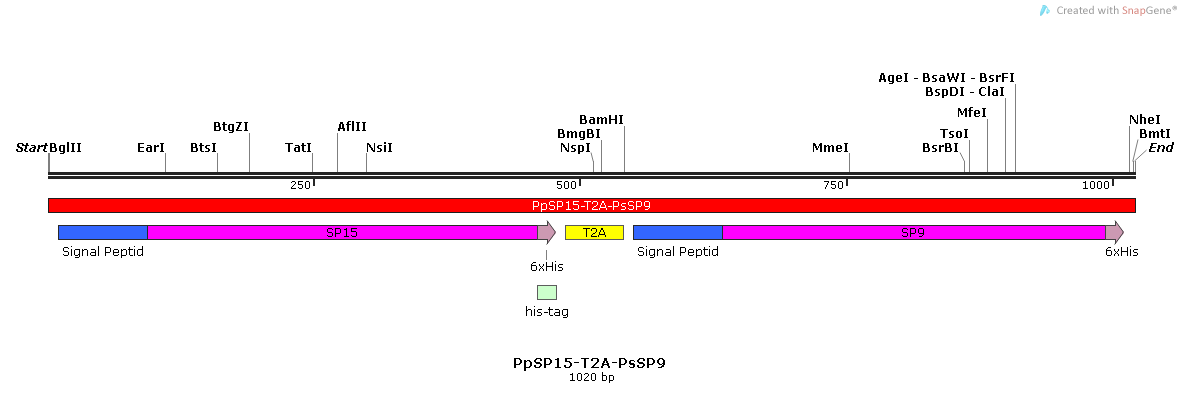


**Graphical abstract**


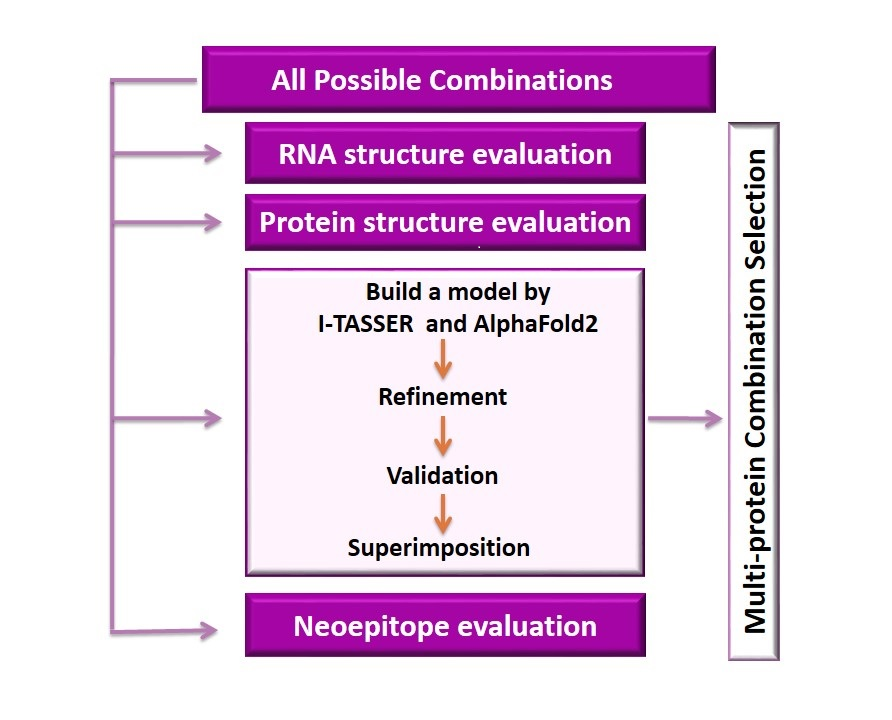

Supplement: Supplementary file 1 — Additional file 1: Table S1. Nucleic acid and protein sequences of all components needed for construct design. Figure S1. Complete sequence of Utr1 and Utr2 of LEXSYcon2 vector. Trans-splicing sites used in mRNA synthesis are highlighted in blue (longest polypyrimidine tract), red (splice acceptor site), and purple (polyA site). Utr1 and Utr2 sequences are obtained from the LEXSYcon2 Expression Kit’s manual (Jena Bioscience). Figure S2. Optimal and centroid secondary structure analysis by RNAfold for all mRNA combinations. The full-sized mRNA sequence of all combinations was submitted to the RNAfold web server for secondary structure prediction. This server predicts two secondary structures, including optimal and centroid structures. a-column: Optimal secondary structure. b-column: Centroid structure. Table S2. Characteristics of Galaxy refined models. Figure S3. Superimposition of all combinations with the original proteins. The validated 3D model of each combination was compared with the original proteins, PpSP15, PsSP9, and PdSP15, using UCSF Chimera v1.15 software. The Superimposition was applied to indicate similarity between two overlaid protein structures by calculating RMSD. For closely homologous proteins, the RMSD is as small as 3 Å. The 3D model color: copper for PpSp15-T2A-PsSp9, PpSp15–PsSp9, PpSP15-Histag-T2A, and PpSP15-Histag. Yellow for PsSP9-T2A-PpSP15, PsSP9–PpSP15, and PsSP9-His-tag-T2A. Red for PpSp15, PsSP9, and PdSP15. Table S3. High-scored HLA-II junctional epitope. Table S4. High scored helper and CTL epitopes of vaccine constituent proteins. Figure S4. Estimation of population coverage of PpSP15 + PsSP9 combined vaccine in the Old World. Population coverage of the predicted epitopes was evaluated with the IEDB population coverage tool. Table S5. Physicochemical characteristics of the vaccine construct compared with original PpSP15 and PsSP9 components. Table S6. Antigenicity and allergenicity of the candidate vaccine construct compared with [file 13071_2022_5437_MOESM1_ESM.docx]
